# Dendritic cell immunoreceptor drives atopic dermatitis by modulating oxidized CaMKII-involved mast cell activation

**DOI:** 10.1172/jci.insight.152559

**Published:** 2022-03-08

**Authors:** Xiaoyan Luo, Jingsi Chen, Huan Yang, Xinyue Hu, Martin P. Alphonse, Yingchun Shen, Yuko Kawakami, Xiaoying Zhou, Wei Tu, Toshiaki Kawakami, Mei Wan, Nathan K. Archer, Hua Wang, Peisong Gao

**Affiliations:** 1 Division of Allergy and Clinical Immunology, Johns Hopkins University School of Medicine, Baltimore, Maryland, USA.; 2 Pediatric Dermatology, Children’s Hospital, Chongqing Medical University, Chongqing, China.; 3 Department of Respiratory Medicine, Xiangya Hospital, Central South University, Changsha, Hunan, China.; 4 Department of Dermatology, Johns Hopkins University School of Medicine, Baltimore, Maryland, USA.; 5 Division of Cell Biology, La Jolla Institute for Allergy and Immunology, La Jolla, California, USA.; 6 Department of Dermatology, School of Medicine, University of California San Diego, La Jolla, California, USA.; 7 Department of Orthopaedic Surgery, Johns Hopkins University School of Medicine, Baltimore, Maryland, USA.

**Keywords:** Dermatology, Immunology, Allergy, Mouse models, Skin

## Abstract

Allergens have been identified as potential triggers in patients with atopic dermatitis (AD). Patients with AD are highly sensitive to cockroach allergen. The underlying mechanism, however, remains undetermined. Here, we established a cockroach allergen–induced AD-like mouse model, and we demonstrate that repeated exposure to cockroach allergen led to aggravated mouse skin inflammation, characterized by increased type 2 immunity, type 2 innate lymphoid cells (ILC2s), and mast cells. Increased mast cells were also observed in patients with AD. Mast cell–deficient mice (*Kit^W-sh/W-sh^*) showed diminished skin inflammation, suggesting that mast cells are required in allergen-induced skin inflammation. Furthermore, DC immunoreceptor (DCIR) is upregulated in skin mast cells of patients with AD and mediates allergen binding and uptake. *DCIR^–/–^* mice or reconstituted *Kit^W-sh/W-sh^* mice with *DCIR^–/–^* mast cells showed a significant reduction in AD-like inflammation. Both in vitro and in vivo analyses demonstrate that *DCIR^–/–^* mast cells had reduced IgE-mediated mast cell activation and passive cutaneous anaphylaxis. Mechanistically, DCIR regulates allergen-induced IgE-mediated mast cell ROS generation and oxidation of calmodulin kinase II (ox-CaMKII). ROS-resistant CaMKII (MM-VV**δ**) prevents allergen-induced mast cell activation and inflammatory mediator release. Our study reveals a DCIR/ROS/CaMKII axis that controls allergen-induced mast cell activation and AD-like inflammation.

## Graphical abstract

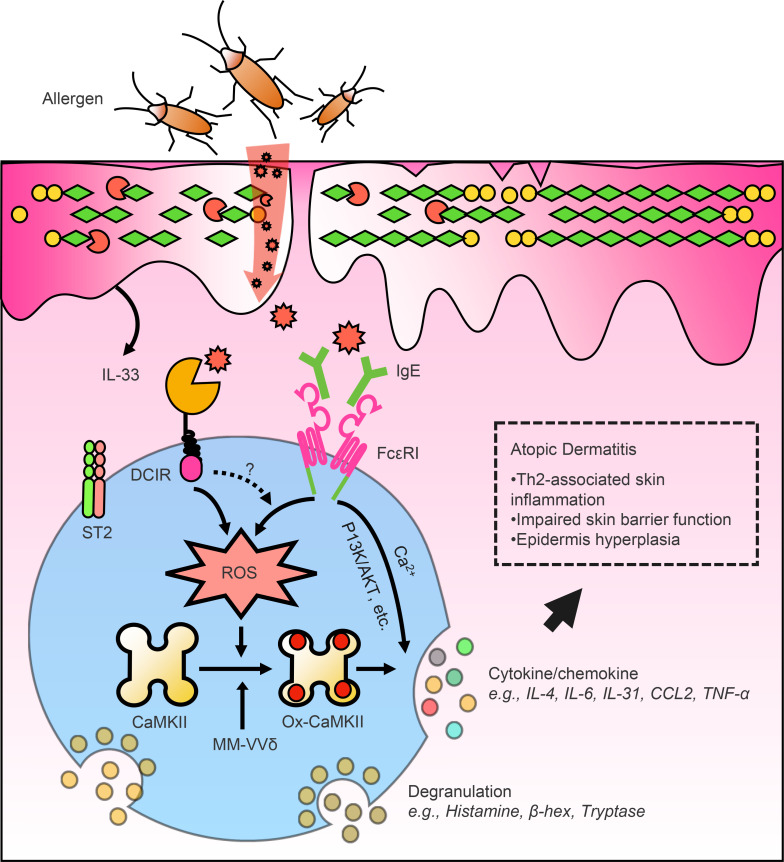

## Introduction

Atopic dermatitis (AD) is a common chronic relapsing inflammatory skin disease that affects 15%–30% of children and approximately 5% of adults in industrialized countries, causing a significant negative impact on the quality of life of patients (1). There has been a 2- to 3-fold increase in pediatric AD over the past several decades (2). Although the etiology of AD is not fully understood, there is growing evidence supporting the role of specific allergens in perpetuating skin inflammation in sensitized patients with AD through impaired skin barrier and inappropriate immune responses to antigens (3, 4). Food and inhalant allergens have been identified as potential trigger factors in sensitized patients with AD (5–7). Cockroach allergen has also been recognized as an important allergen associated with allergic diseases (8). Most patients with AD are highly sensitive to cockroach allergen (9–12). However, little is known about how cockroach allergen triggers AD and its underlying mechanisms.

Most allergens contain complex glycan modifications attached to glycoproteins and glycolipids that are important in allergen-induced allergic responses (8, 13, 14). We have demonstrated that cockroach allergen contains glycans, many of which are mannose terminated, which are major determinants in allergic immune responses (13, 15). C-type lectin receptors (CLRs) are crucial in the recognition of complex glycan structures on various pathogens and in facilitating pathogen endocytosis and presentation (16–18). Of these, DC immunoreceptor (DCIR) is one of the major CLRs in DCs. It has a carbohydrate recognition domain in its extracellular portion and an immunoreceptor tyrosine-based inhibitory motif (ITIM) in its cytoplasmic tail (19, 20). DCIR has been implicated in antigen processing and presentation and allergen-induced inhibitory or active signaling (13, 21–24), and it has been associated with development of tuberculosis (24) and autoimmune diseases (25). These findings highlight the significance of DCIR in allergen-induced diseases like AD.

Mast cells are known to be critical in the regulation of allergic diseases because of their preferential localization close to skin epidermis or at the site of the tissue mucosa where exposure to environmental antigens and chemicals often occurs (26–30). Mast cells have large numbers of granules, containing mediators like histamine, serotonin, proteases, and cytokines, and they can synthesize prostaglandins, leukotrienes, and cytokines, thereby initiating inflammatory immune responses (31). Increased numbers of mast cells have been observed in the skin lesions of patients with AD (32, 33) or AD mouse models (34, 35). It has been suggested that mast cells are involved in complex cellular networks that maintain skin barrier function and homeostasis (36, 37). Mast cells have been shown to mediate the allergic responses through the IgE/FcεRI complex (38) and/or IgE-independent mechanisms (39–42) upon allergen exposure. Our recent evidence suggests that DCIR is expressed in basophils and involved in cockroach allergen binding and uptake by basophils (13). This led us to investigate whether DCIR is expressed in mast cells and whether it modulates mast cell activation and allergen-induced skin allergic inflammation.

Here, we report that repeated exposure to cockroach allergen can induce AD-like skin inflammation and that mast cells are required in this pathological process. Further studies address that DCIR, particularly the DCIR in mast cells, plays an important role in allergic skin inflammation and allergen-induced anaphylaxis. Our in vitro analyses suggest that DCIR participates in allergen binding and uptake in mast cells and regulates allergen-induced mast cell activation. Furthermore, we reveal that DCIR regulates allergen-induced and IgE-mediated mast cell activation through controlling ROS generation and oxidative activation of CaMKII. Most importantly, mast cells with ROS-resistant CaMKII showed protection against allergen-induced cell activation and inflammatory mediator release. Thus, targeting the functional axis of DCIR-CaMKII responsible for the mast cell activation and mediator release may be of therapeutic benefit to patients with AD.

## Results

### Cockroach allergen exposure induces AD-like skin inflammation.

**Figure 1:**
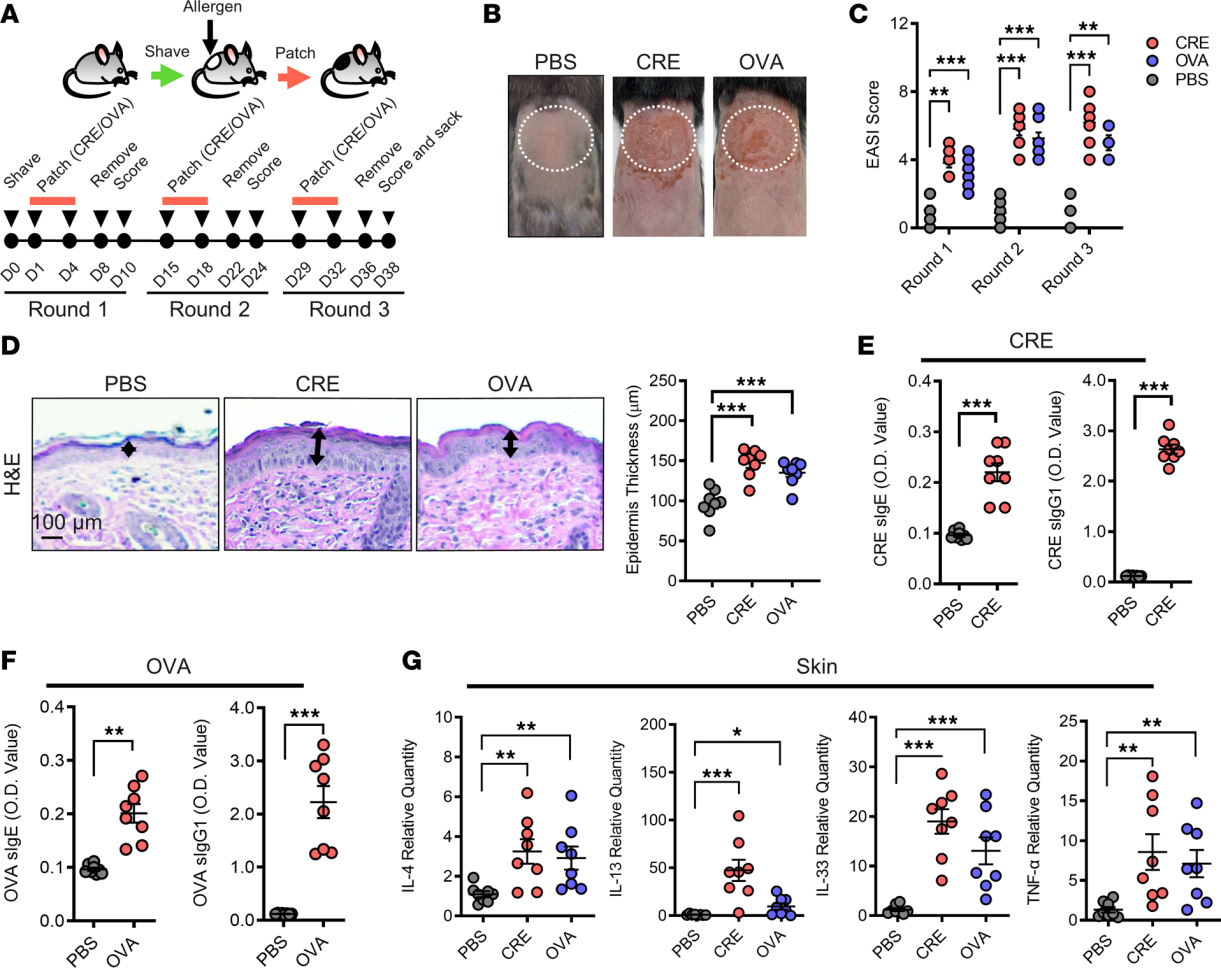
Clinical features of cockroach allergen exposure–induced AD-like skin inflammation. (**A**) Schematic of experimental protocol for the generation of cockroach allergen–induced AD mouse model. (**B**) Representative skin images of mice exposed to PBS, CRE, and OVA. (**C**) Severity of clinical signs (e.g., bleeding, eruption, scaling) as assessed by EASI score (0, no symptom; 1, mild; 2, intermediate; 3, severe) on day 10 (round 1), day 24 (round 2), and day 38 (round 3). (**D**) Representative H&E staining and epidermal thickness (μm) of skin tissues. Scale bar: 100 μm. (**E** and **F**) Serum levels of specific IgE and IgG1 to CRE (**E**) or OVA (**F**). (**G**) Quantitative PCR analysis of IL-4, IL-13, IL-33, and TNF-α expression in the skin tissues of PBS-, CRE-, or OVA-treated mice. Each circle represents 1 mouse. *n* = 8. (**C**–**G**) Data represent mean ± SEM of 2 independent experiments. Data in **C**, **D**, and **G** were compared by 2-way ANOVA. Data in **E** and **F** were compared using a 2-tailed Student’s *t* test. *P < 0.05, **P < 0.01, and ***P < 0.001.

We first examined whether repetitive topical exposure to cockroach allergen can induce AD-like skin inflammation in a cockroach allergen–induced mouse model of AD modified from previous reports (Figure 1A) (43–45). Cockroach allergen challenge was performed by epicutaneous treatment with 100 μg of cockroach extract (CRE). Mice were also challenged with either 100 μg of OVA or saline as a positive or negative control, respectively. Similar to OVA, CRE-treated mice developed skin inflammation, including enhanced erythema/hemorrhage, eruption, and scarring/dryness, as compared with PBS-treated mice (Figure 1B). The dermatitis was further supported by Eczema Area and Severity Index (EASI) score, a method for quantifying the severity of clinical signs (46, 47), on day 2 after every patch removal (Figure 1C). Consistently, histological analysis showed thickening of the epidermal and dermal layers, with marked infiltration of leukocytes in the dermis (Figure 1D). Furthermore, higher serum levels of allergen-specific IgE (sIgE) and IgG1 (sIgG1) over controls were observed for mice with epicutaneous sensitization to CRE (Figure 1E) or OVA (Figure 1F), respectively. In agreement with these findings, skin tissues of CRE- or OVA-sensitized mice displayed increased inflammatory cytokine transcripts, including IL-4, IL-13, IL-33, and TNF-α (Figure 1G). These data indicate that epicutaneous sensitization with cockroach allergen can induce AD-like skin inflammation.

### Increased Th2 cells, type 2 innate lymphoid cells (ILC2s), and mast cells in the lesional skins of AD mouse model.

**Figure 2:**
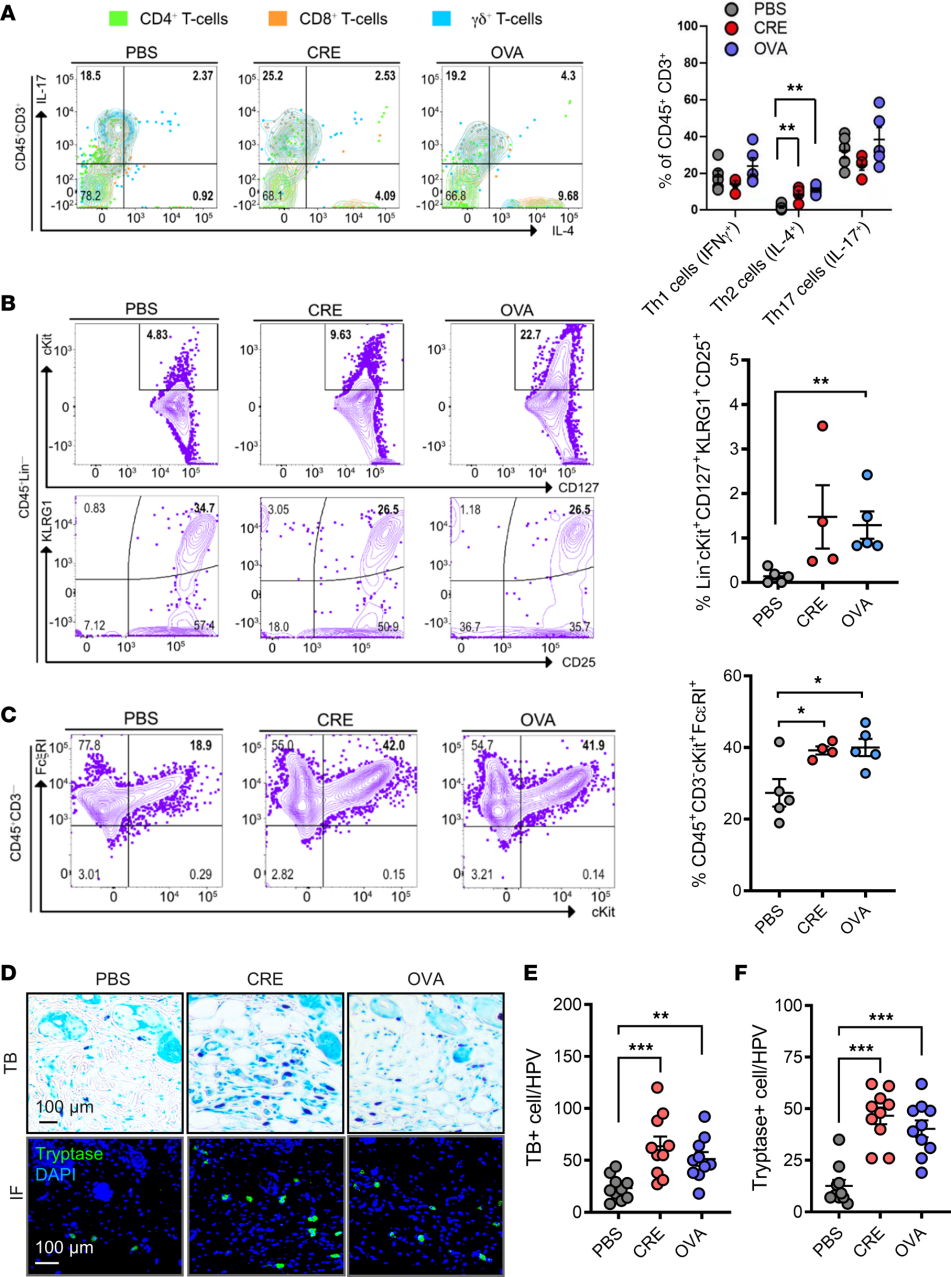
Increased Th2. **ILC2, and mast cells in the lesional skins of AD mouse model**. (**A**) Representative flow cytometry plots for Th2 (IL-4^+^) and Th17 (IL-17^+^) overlaid with expression of CD4^+^ T cells (CD45^+^CD3^+^CD4^+^CD8^–^γδTCR^–^), CD8^+^ T cells (CD45^+^CD3^+^CD8^+^), and γδ T cells (CD45^+^CD3^+^CD8^–^γδTCR^+^) and percentage of Th1 cells (IFN-γ^+^), Th2 (IL-4^+^), and Th17 (IL-17^+^) populations in the lesional skins of AD mouse model. (**B**) Representative flow cytometry plots for ILC2s (CD45^+^Lin^–^KLRG1^+^CD127^+^CD25^+^ cells) and percentage of ILC2s in the lesional skins of AD mouse model. (**C**) Representative flow cytometry plots for mast cells (CD45^+^CD3^–^cKit^+^FcεRI^+^) and percentage of mast cells in the lesional skins of AD mouse model. (**D**) Representative Toluidine blue (upper panel, blue) and tryptase (lower panel, green) staining of skin tissue sections from vehicle-, CRE-, or OVA-treated mice. Scale bar: 100 μm. (**E** and **F**) Quantification of cells with positive staining for Toluidine blue (**E**) and tryptase (**F**) in **D**. *n* = 10. Data represent mean ± SEM. Data were compared by 2-way ANOVA. *P < 0.05, **P < 0.01, and ***P < 0.001.

To further characterize the cockroach allergen–induced skin inflammation, percentages of T cells, ILC2, and mast cells from biopsies of the lesional skins of CRE-treated or untreated mice were evaluated by using flow cytometry as previously described (48). The gating strategy for the flow cytometry analysis is provided in Supplemental Figure 1, A and B (supplemental material available online with this article; https://doi.org/10.1172/jci.insight.152559DS1). Compared with those untreated mice, CRE- or OVA-treated mice showed significantly increased percentages of skin Th2 (IL-4^+^) cells (Figure 2A). In contrast, no statistical differences were observed for the percentages of skin Th1 (IFN-γ^+^) and Th17 (IL-17^+^) cells. ILC2 present in the skin have recently emerged as important contributors to skin inflammation (49). Thus, we detected ILC2 cells (CD45^+^Lin^–^KLRG1^+^CD127^+^CD25^+^) in the skin of the allergen-induced AD mouse model. As expected, ILC2 cells were clearly increased in CRE- or OVA-treated mice relative to those untreated mice (Figure 2B), while the increase did not reach statistical significance for CRE treatment because of the limited sample size (*P* = 0.071). Studies have also provided evidence that mast cells were increased in skin lesions of patients with AD (32, 33) and have suggested that mast cells may participate in maintaining barrier function and homeostasis (30, 36, 37). Thus, we specifically analyzed mast cells (CD45^+^CD3^–^FcεRI^+^cKit^+^ cells) in the skin isolated from those CRE- or OVA-treated mice (Figure 2C). Compared with those untreated mice, CRE- or OVA-treated mice showed significantly increased skin mast cells. The increased mast cells were further confirmed by both Toluidine blue (TB) staining (Figure 2, D and E) and immunofluorescence staining with tryptase (Figure 2, D and F), a marker generally reflecting the population of total active mast cells. Most importantly, we analyzed mast cell infiltrates of lesional skin collected from patients with AD and healthy individuals. The clinical and demographic data of patients with AD and healthy control subjects were included in Supplemental Tables 1 and 2. Skin samples from patients with AD showed increased epidermal thickness compared with those from healthy controls (Supplemental Figure 2, A and C). Notably, these skin tissues from patients with AD showed increased mast cells in the dermis as assessed by TB staining (Supplemental Figure 2, B and D). Collectively, these findings suggest increased Th2, ILC2s, and mast cells in the lesional skins of AD.

### Mast cells are required in cockroach allergen–induced allergic skin inflammation.

**Figure 3:**
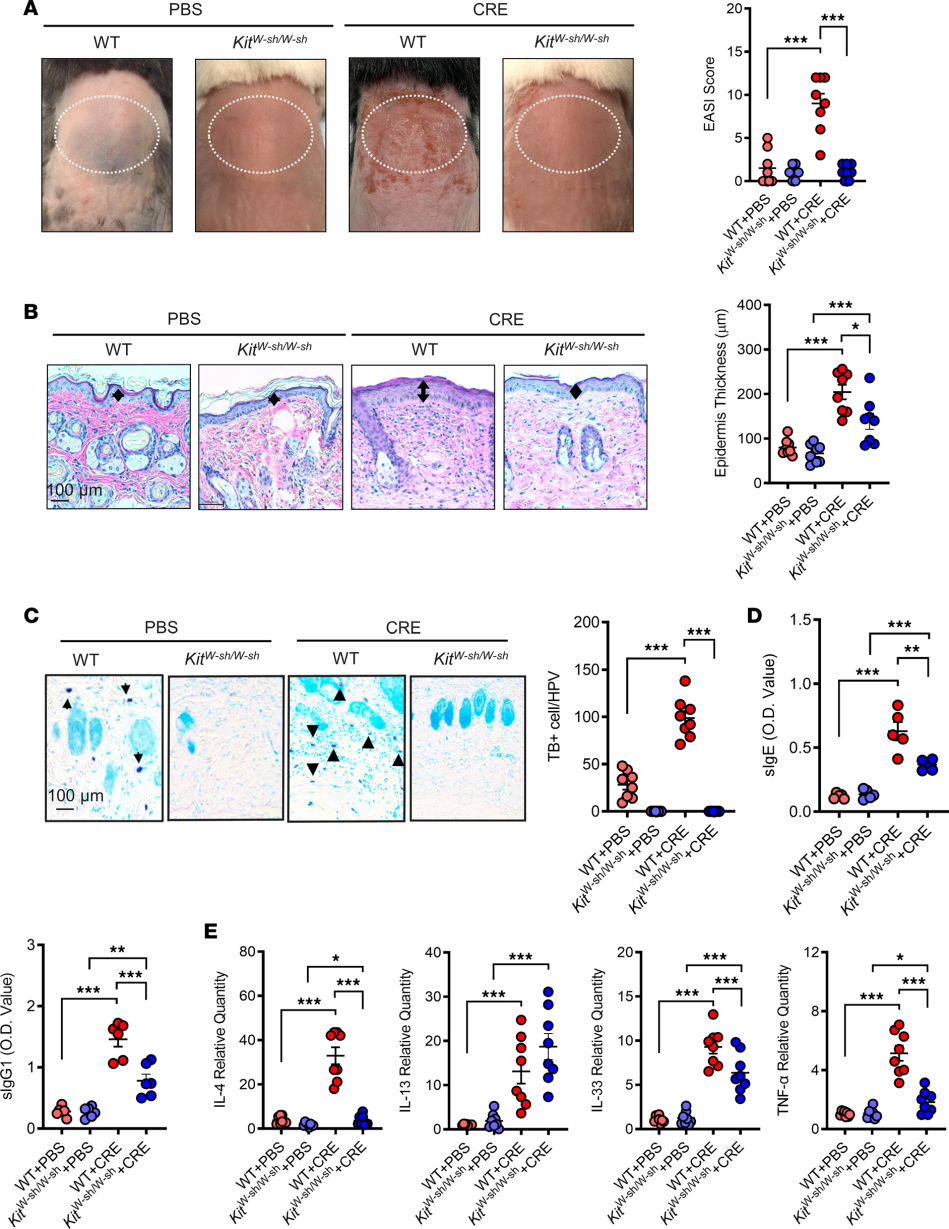
Mast cells are required in cockroach allergen–induced allergic skin inflammation. (**A**) Representative skin images and EASI scores of PBS- and CRE-treated WT and *Kit^W-sh/W-sh^* mice. (**B**) Representative H&E staining and epidermal thickness (μm) of skin tissues of PBS- and CRE-treated WT and *Kit^W-sh/W-sh^* mice. (**C**) Representative Toluidine blue staining and quantification of cells with positive staining for Toluidine blue of skin tissue sections of PBS- and CRE-treated WT and *Kit^W-sh/W-sh^* mice. Scale bar: 100 μm. Arrows represent mast cells. (**D**) Serum levels of specific IgE and IgG1 to CRE. (**E**) Quantitative PCR analysis of IL-4, IL-13, IL-33, and TNF-α expression in the skin tissues of PBS- and CRE-treated WT and *Kit^W-sh/W-sh^* mice. Each circle represents 1 mouse. *n* = 6–8. Data represent mean ± SEM of 2 independent experiments. Data were compared by 2-way ANOVA. *P < 0.05, **P < 0.01, ***P < 0.001.

Next, we determined whether the increased mast cells are required in the pathogenesis of cockroach allergen–induced mouse model of AD by using the mast cell–deficient mice (*Kit^W-sh/W-sh^*) (50–52). As noted, *Kit^W-sh/W-sh^* mice showed complete protection against cockroach allergen–induced erythema/hemorrhage, eruption, and scarring/dryness (EASI score, Figure 3A) and epidermal hyperplasia (Figure 3B). Furthermore, histological analysis with TB staining confirmed mast cell deficiency in *Kit^W-sh/W-sh^* mice but increased in the lesional skin of WT mice after CRE treatment (Figure 3C). *Kit^W-sh/W-sh^* mice also showed significantly lower levels of sIgE and sIgG1 in serum (Figure 3D) and reduced expression of IL-4, IL-33, and TNF-α in the lesional skin tissues as compared with WT mice (Figure 3E). No significant change was noted for IL-13. This finding suggests that mast cells are required in cockroach allergen–induced skin inflammation. We further determined whether IgE plays a role in CRE-mediated AD-like inflammation by using IgE-deficient mice (IgE-KO). While no allergen-specific IgE was detected in serum of cockroach allergen-treated IgE-KO mice (Supplemental Figure 3A), these mice showed increased allergen-specific IgG1 (Supplemental Figure 3A), skin epithelial thickness (Supplemental Figure 3, B and C), and Th2 cytokine expression (Supplemental Figure 3, D and E). Interestingly, relative to WT mice, IgE-KO mice showed comparable levels of cockroach allergen–induced allergen-specific IgG1, skin epithelial thickness, and Th2 cytokine expression. These findings suggest that other factors the beyond IgE-mediated pathway may play critical roles in cockroach allergen–induced mast cell activation and AD.

### DCIR is highly expressed in skin mast cells of patients with AD and mouse model with AD, and it mediates cockroach allergen binding and uptake.

**Figure 4:**
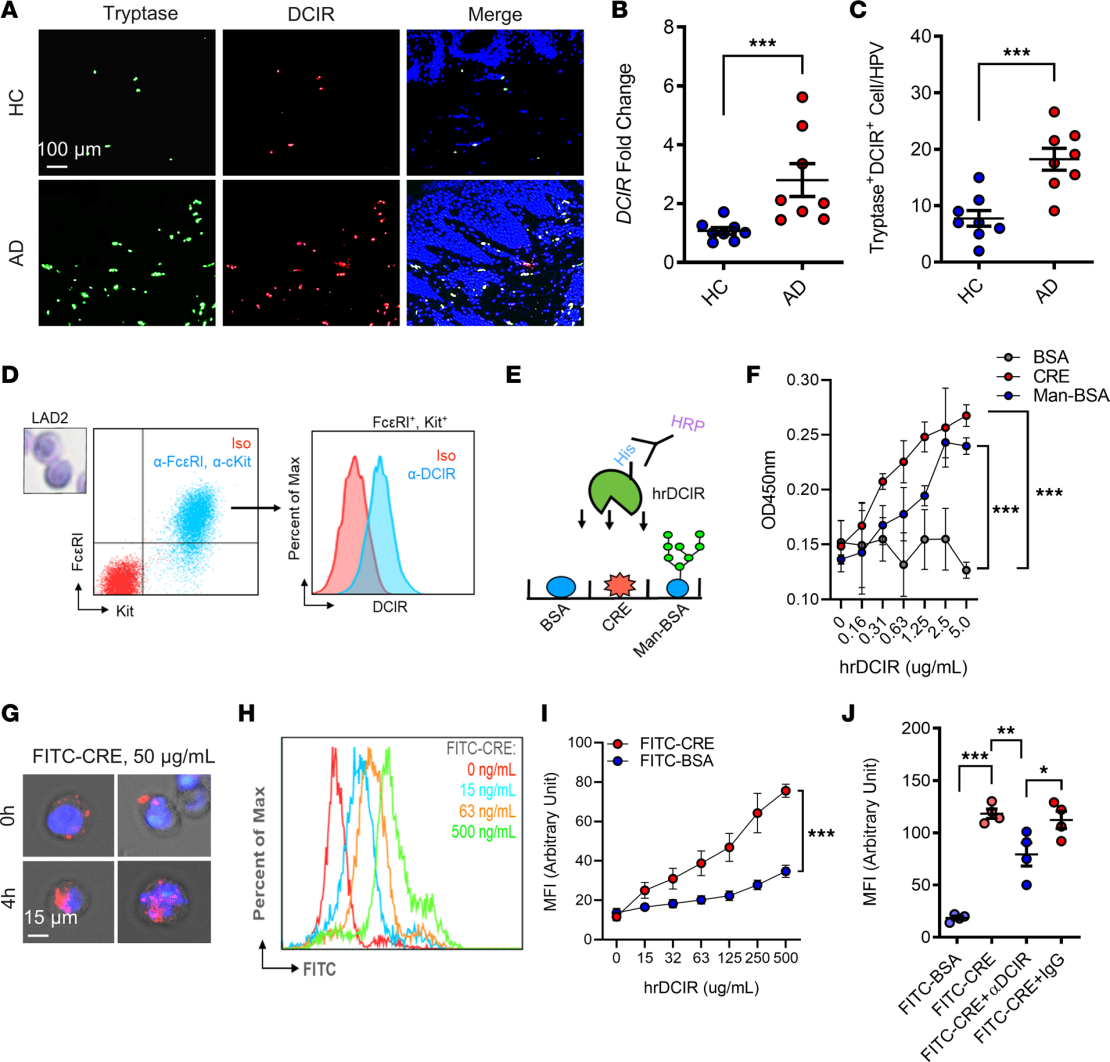
DCIR mediates cockroach allergen binding and uptake. (**A** and **B**) Representative immunofluorescence images of dorsal skin sections and fluorescence analysis of DCIR staining in the skin tissues of patients with AD and controls (*n* = 8). Scale bar: 100 μm. (**C**) Quantification analysis of *DCIR^+^tryptase^+^* cells in the lesion skin of patients with AD and controls. (**D**) Flow cytometry analysis of DCIR expression in human mast cell line *cKit^+^FcεRI^+^* LAD2 cells. (**E**) Scheme of experimental protocol for the direct bindings of human recombinant DCIR (hrDCIR) to BSA, CRE, and Man-BSA. (**F**) Direct binding of different doses of hrDCIR (0–5.0 μg/mL) to BSA, CRE, or Man-BSA (*n* = 3). (**G**) Representative immunofluorescence images of FITC-CRE uptake by LAD2. Scale bar: 15 μm. (**H** and **I**) Flow cytometry analysis (**H**) and quantification (**I**) of FITC-CRE uptake at different doses (1–500 ng/mL) by LAD2 cells (*n* = 3). (**J**) Inhibition of FITC-CRE uptake in LAD2 cells pretreated with DCIR neutralizing antibody (α-DCIR) or IgG isotype (*n* = 4). Data represent mean ± SEM of 2 independent experiments. Data in **B**, **C**, and **I** were compared using a 2-tailed Student’s *t* test. Data in **F** and **J** were compared by 2-way ANOVA. *P < 0.05, **P < 0.01, ***P < 0.001.

Our previous study has demonstrated that DCIR is highly expressed in basophils (13). Thus, we detected DCIR expression in skin mast cells of patients with AD. Histological analysis showed that skin tissues from patients with AD have increased expression of DCIR as compared with healthy controls (Figure 4, A and B). As expected, DCIR was highly expressed in mast cells as determined by coimmunofluorescence staining for DCIR and tryptase (Figure 4, A and C). The increased DCIR in skin tissues of patients with AD was also confirmed by reverse transcription PCR (RT-PCR) (Supplemental Figure 2E). Increased DCIR in mast cells was also confirmed by in vitro flow cytometry analysis with FcεRI^+^cKit^+^ LAD2 cells, a human mast cell line (Figure 4D). The increased expression of DCIR in the human skin tissues was also supported by the findings in our mouse model by using immunofluorescence staining (Supplemental Figure 4, A and B) and RT-PCR (Supplemental Figure 4C). Furthermore, the increased DCIR observed was significantly attenuated in skin tissues of mast cell–deficient *Kit^W-sh/W-sh^* mice as compared with those from WT mice (Supplemental Figure 4, A–C). Next, we investigated whether DCIR in mast cells, similar to that in basophils (13), is involved in allergen binding and uptake as illustrated in Figure 4E. Briefly, 20 μg of CRE was incubated with different doses of purified recombinant human DCIR-His Tag (hrDCIR, 0–5.0 μg/mL) for 30 minutes at room temperature (RT). Equal amount of Man-BSA and BSA was used as a positive and negative control, respectively. The binding of CRE-DCIR was then detected by ELISA. Consistent with the mannosylated-BSA (Man-BSA), CRE showed a dose-dependent binding to DCIR (Figure 4F), indicating that cockroach allergen contains a natural ligand for DCIR that can bind DCIR. Next, we tested whether DCIR in mast cells is involved in cockroach allergen uptake. LAD2 cells were treated with FITC-labeled CRE (FITC-CRE), and uptake was detected by immunostaining (Figure 4G) and flow cytometry analysis (Figure 4H). FITC-CRE, but not FITC-BSA, showed a dose-dependent uptake by LAD2 cells (Figure 4I). To further determine the role of DCIR in FITC-CRE uptake, LAD2 cells were pretreated with α-DCIR antibody or IgG isotype control for 30 minutes before treatment with FITC-CRE or FITC-BSA. Compared with IgG control, α-DCIR significantly inhibited LAD2 cell FITC-CRE uptake (Figure 4J). Taken together, our in vitro data indicate that DCIR is highly expressed in mast cells and plays a role in cockroach allergen binding and uptake.

### Lack of DCIR protects against cockroach allergen–induced skin allergic inflammation.

**Figure 5:**
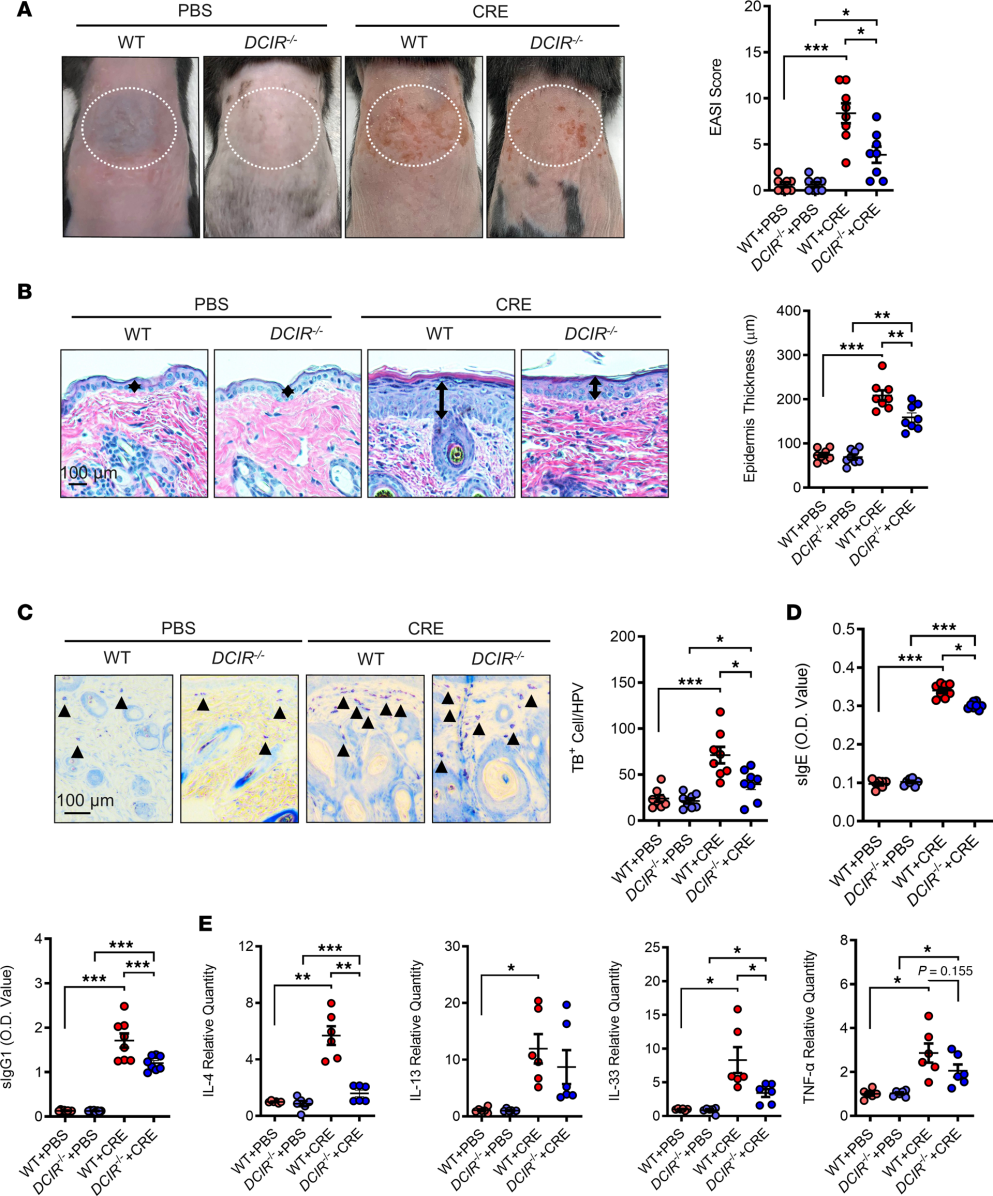
Lack of DCIR protects against cockroach allergen–induced skin allergic inflammation. (**A**) Representative skin images and EASI scores of PBS- and CRE-treated WT and DCIR^–/–^ mice. (**B**) Representative H&E staining and epidermal thickness (μm) of skin tissues of PBS- and CRE-treated WT and *DCIR^–/–^* mice. (**C**) Representative Toluidine blue staining and quantification of cells with positive staining for Toluidine blue of skin tissue sections of PBS- and CRE-treated WT and *DCIR^–/–^* mice. Scale bar: 100 μm. Arrows represent mast cells. (**D**) Serum levels of specific IgE and IgG1 to CRE. (**E**) Quantitative PCR analysis of IL-4, IL-13, IL-33, and TNF-α expression in the skin tissues of PBS- and CRE-treated WT and *DCIR^–/–^* mice. Each circle represents 1 mouse. *n* = 8. Data represent mean ± SEM of 2 independent experiments. Data were compared by 2-way ANOVA. *P < 0.05, **P < 0.01, ***P < 0.001.

To define the role of DCIR in the pathogenesis of AD, we used mice lacking DCIR (*DCIR^–/–^*) to generate a cockroach allergen–challenged mouse model of AD following the protocol illustrated in Figure 1A. Compared with WT mice, *DCIR^–/–^* mice exposed to cockroach allergen revealed the attenuation of allergen-induced skin inflammation, as defined by EASI score (Figure 5A) and histological analysis of epidermal thickness (Figure 5B). In line with these observations, *DCIR^–/–^* mice with epicutaneous sensitization of CRE had a reduced number of skin mast cells (Figure 5C), lower levels of sIgE and sIgG1 in serum (Figure 5D), and attenuated expression of Th2 cytokine IL-4 and skin epithelial cell–derived cytokine IL-33 in the skin tissues (Figure 5E). No significant changes were observed for IL-13 and TNF-α. These data suggest that DCIR may play a critical role in cockroach allergen–induced skin inflammation.

### Adoptive transfer of DCIR^+^, but not DCIR^–^ mast cells, fully resumed AD in Kit^W-sh/W-sh^ mice.

**Figure 6:**
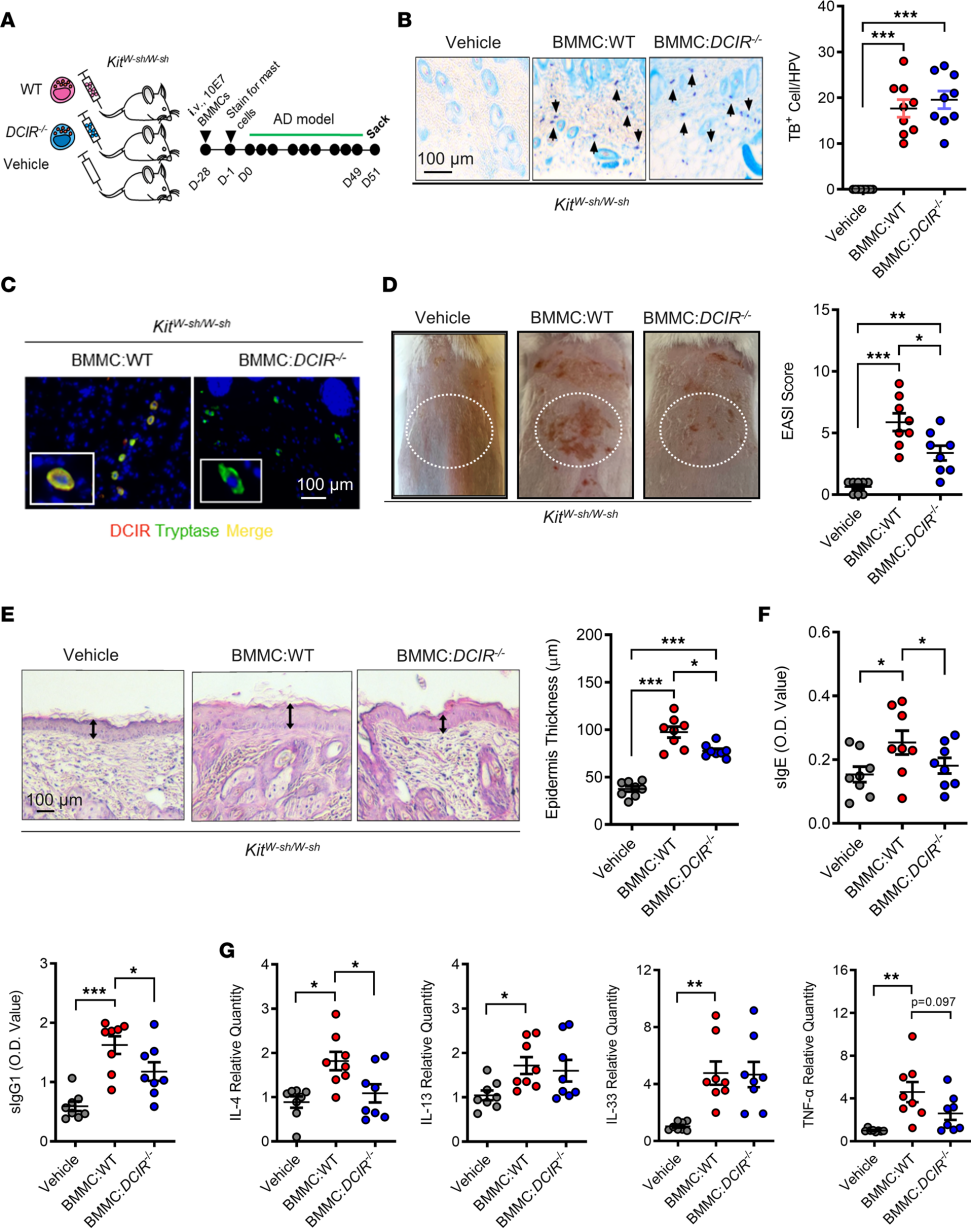
I.v. transfer of *DCIR^+^* mast cells resumes AD in ***Kit^W-sh/W-sh^*** mice. (**A**) Scheme of experimental protocol of i.v. transfer of DCIR^+^ versus DCIR^–^ mast cells into *Kit^W-sh/W-sh^* mice for the generation of AD mouse model. (**B**) Representative Toluidine blue staining and quantification of cells with positive staining for Toluidine blue of skin tissue sections of *Kit^W-sh/W-sh^* mice with *DCIR^+^* or *DCIR^–^* mast cells (*n* = 9). (**C**) Representative immunofluorescence images of mast cells with (yellow) or without (blue) DCIR expression. (**D**) Representative skin images and EASI scores of *Kit^W-sh/W-sh^* mice with *DCIR^+^* or *DCIR^–^* mast cells (*n* = 8). (**E**) Representative H&E staining and epidermal thickness (μm) of skin tissues of *Kit^W-sh/W-sh^* mice with *DCIR^+^* or *DCIR^–^* mast cells (*n* = 8). Scale bar: 100 μm. Arrows represent mast cells. (**F**) Serum levels of specific IgE and IgG1 to CRE (*n* = 8). (**G**) Quantitative PCR analysis of IL-4, IL-13, IL-33, and TNF-α expression in the skin tissues of *Kit^W-sh/W-sh^* mice with *DCIR^+^* or *DCIR^–^* mast cells. Each circle represents 1 mouse (*n* = 8). Data represent mean ± SEM of 2 independent experiments. Data were compared by 2-way ANOVA. *P < 0.05, **P < 0.01, ***P < 0.001.

*DCIR^+^tryptase^+^* mast cells were observed in the skin tissues of patients with AD and mouse model. This led us to postulate that the attenuated skin inflammation noted in *DCIR^–/–^* mice may be due to the DCIR deficiency specifically in mast cells. Thus, we reconstituted *Kit^W-sh/W-sh^* mice with BMMCs from WT and *DCIR^–/–^* mice via tail vein following the protocol as illustrated in Figure 6A. After 4 weeks, these reconstituted mice were used to generate cockroach allergen–induced AD models as previously illustrated in Figure 1A. The reconstituted mast cells in the lesional skin of the AD mouse model were confirmed by TB staining (Figure 6B). Mast cells in *Kit^W-sh/W-sh^* mice reconstituted with WT BMMCs are comparable with those with *DCIR^–/–^* BMMCs. Furthermore, DCIR were found only in the lesional skin of *Kit^W-sh/W-sh^* mice reconstituted with WT BMMCs — not in those with *DCIR^–/–^* BMMCs (Figure 6C). As expected, *Kit^W-sh/W-sh^* mice reconstituted with *DCIR^–/–^* BMMCs showed attenuated skin inflammation and lower EASI scores as compared with those reconstituted with WT BMMCs (Figure 6D). The results were further supported by histological analysis of epidermal thickness (Figure 6E). Furthermore, these mice reconstituted with *DCIR^–/–^* BMMCs showed significantly lower levels of serum sIgE and sIgG1 (Figure 6F) and reduced expression of IL-4 in the skin tissues (Figure 6G). Collectively, these data indicate that DCIR in mast cells plays a critical role in cockroach allergen–induced skin allergic inflammation.

### DCIR regulates IgE-mediated mast cell activation and allergic responses.

**Figure 7:**
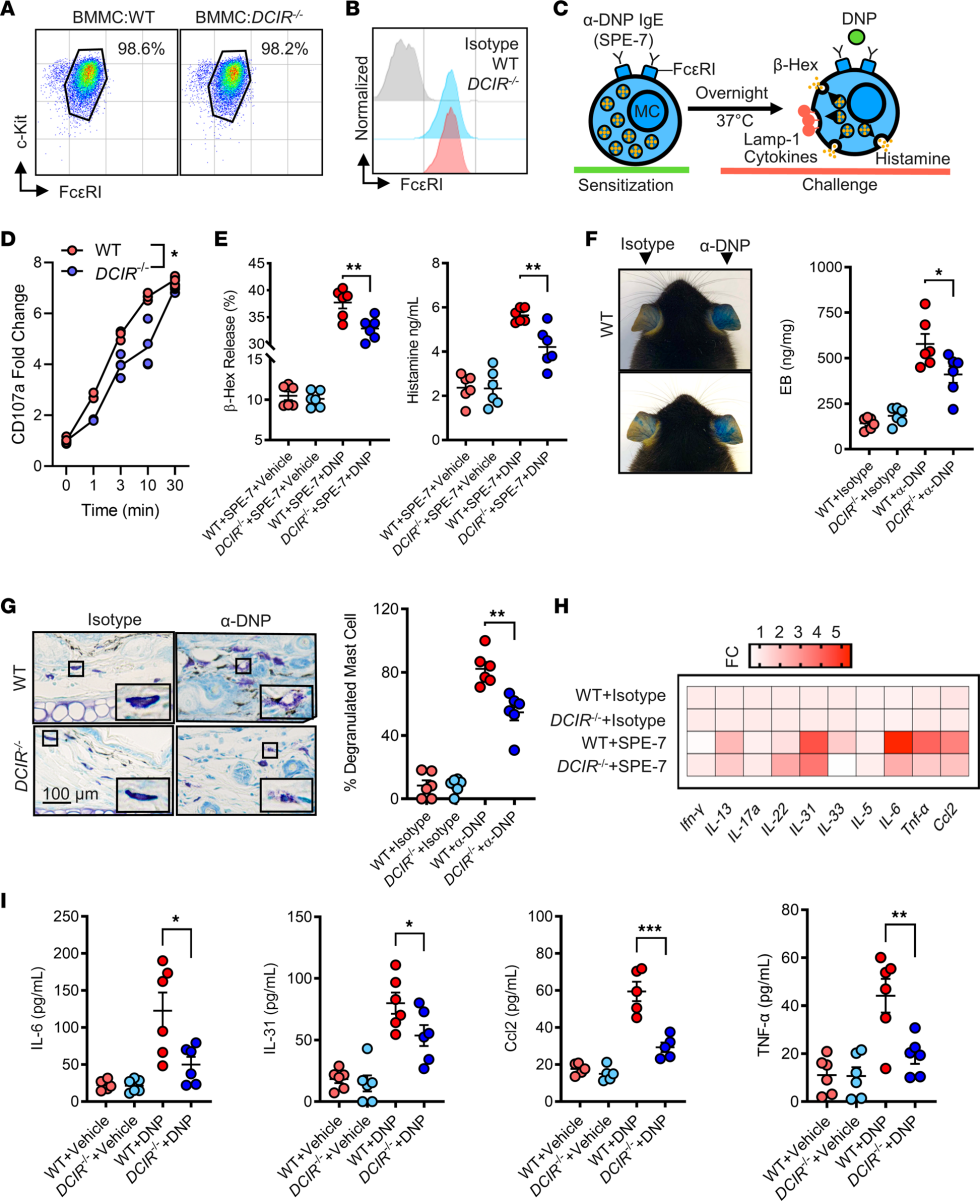
DCIR regulates IgE-mediated mast cell activation and allergic responses. (**A** and **B**) BMMCs were confirmed and FcεRI expression was detected in BMMCs from WT and *DCIR^–/–^* mice by flow cytometry analysis. (**C**) Scheme of experimental protocol for IgE-mediated mast cell activation. (**D**) Flow cytometry analysis of CD107a expression in DNP-activated mast cells at different time points (*n* = 3). (**E**) ELISA analyses of β-hexosaminidase and histamine levels in supernatants of DNP-activated BMMCs from WT and *DCIR^–/–^* mice (*n* = 6). (**F**) Representative images of Evans blue–stained extravasation into ear skin of DNP-treated WT and *DCIR^–/–^* mice and quantification of the extravasation of Evans blue leakage into the skin (*n* = 6). (**G**) Representative Toluidine blue staining and quantification of cells with positive staining for Toluidine blue of skin tissue sections of DNP-treated WT and *DCIR^–/–^* mice (*n* = 6). Scale bar: 100 μm. (**H**) Heatmap of fold changes relative to the WT untreated group for the multiplex assays of cytokines and chemokines in supernatants of BMMCs from WT and *DCIR^–/–^* mice (*n* = 6). (**I**) ELISA analyses of cytokines and chemokines in supernatants of BMMCs from WT and *DCIR^–/–^* mice (*n* = 6). Data represent mean ± SEM of 2 independent experiments. Data were compared using a 2-tailed Student’s *t* test. *P < 0.05, **P < 0.01, ***P < 0.001.

To explore the role of DCIR in mast cells in regulating allergic skin inflammation, we investigated whether DCIR modulates allergen-induced, IgE-mediated mast cell activation. BMMCs were isolated from WT and *DCIR^–/–^* mice and cultured as previously described (53–55). Mast cell phenotype was confirmed by flow cytometry analysis with antibodies against cKit and FcεRI (Figure 7A). No difference was observed for FcεRI in BMMCs from WT and *DCIR^–/–^* mice (Figure 7B). Following the protocol for in vitro analysis as illustrated in Figure 7C, BMMCs were sensitized with 0.5 μg/mL anti–DNP-IgE overnight at 37°C and then challenged with 100 ng/mL of DNP-HSA for 30 minutes at 37°C. Mast cell activation was assessed by the expression of surface activation marker CD107a (LAMP-1), functional assays (e.g., β-hexosaminidase, histamine release), and cytokine release. Flow cytometry analysis demonstrated that BMMCs from *DCIR^–/–^* mice showed a marked reduction in the expression of CD107a (LAMP-1) after DNP sensitization and challenge as compared with those from WT mice (Figure 7D). The same pattern was observed for mast cell degranulation as assessed by β-hexosaminidase and histamine release (Figure 7E). The results were also confirmed in OVA-sensitized and -challenged human mast cells with or without DCIR knockdown. DCIR in LAD2 cells was knocked down by siRNA and then confirmed by RT-PCR (Supplemental Figure 5A). Compared with LAD2 cells, LAD2 cells with DCIR knockdown showed reduced expression of LAMP-1 (Supplemental Figure 5B) and lower levels of β-hexosaminidase (Supplemental Figure 5C), IL-5, and IL-13 (Supplemental Figure 5D) after treatment with OVA. To further determine whether DCIR plays a role in mast cell degranulation in vivo, we sensitized WT and *DCIR^–/–^* mice by intradermal injection of 100 ng anti–DNP-IgE in the skin of a mouse ear and then challenged them by the injection of 200 μg DNP-HSA in Evan’s blue dye via tail vein (Supplemental Figure 6A). Mast cell degranulation was monitored by the vascular leakage of Evan’s blue dye using the passive cutaneous anaphylaxis (PCA) assay. Both WT and *DCIR^–/–^* mice showed Evan’s blue dye leaking in ears, but *DCIR^–/–^* mice showed much less leaking than WT mice (Figure 7F). Furthermore, both WT and *DCIR^–/–^* mice showed an increased percentage of degranulated mast cells among total mast cells in ears upon the sensitization and challenge of DNP; however, *DCIR^–/–^* mice displayed much less than WT mice (Figure 7G). Similar results were found in CRE-treated mice for the vascular leakage of Evan’s blue dye cells (Supplemental Figure 6B) and percentage of degranulated mast cells (Supplemental Figure 6C). Additionally, we measured inflammatory mediators in supernatants with the MSD U-PLEX Biomarker Group 1 (Mouse) Multiplex Assays with a total of 10 highly selected cytokines/chemokine (IL-5, IL-6, IL-13, IL-17A, IL-22, IL-31, IL-33, IFN-γ, TNF-α, and CCL2) (Figure 7H). Significant increases were observed for IL-6, IL-13, IL-31, TNF-α, and CCL2 in supernatants of DNP-treated BMMCs. Of these, IL-6, IL-31, TNF-α, and CCL2 were much lower in DNP-treated *DCIR^–/–^* BMMCs than WT BMMCs (Figure 7I). Collectively, these data suggest that DCIR may play a critical role in IgE-mediated mast cell activation and proinflammatory mediator release.

### DCIR modulates allergen-induced mast cell ROS generation and CaMKII oxidation.

**Figure 8:**
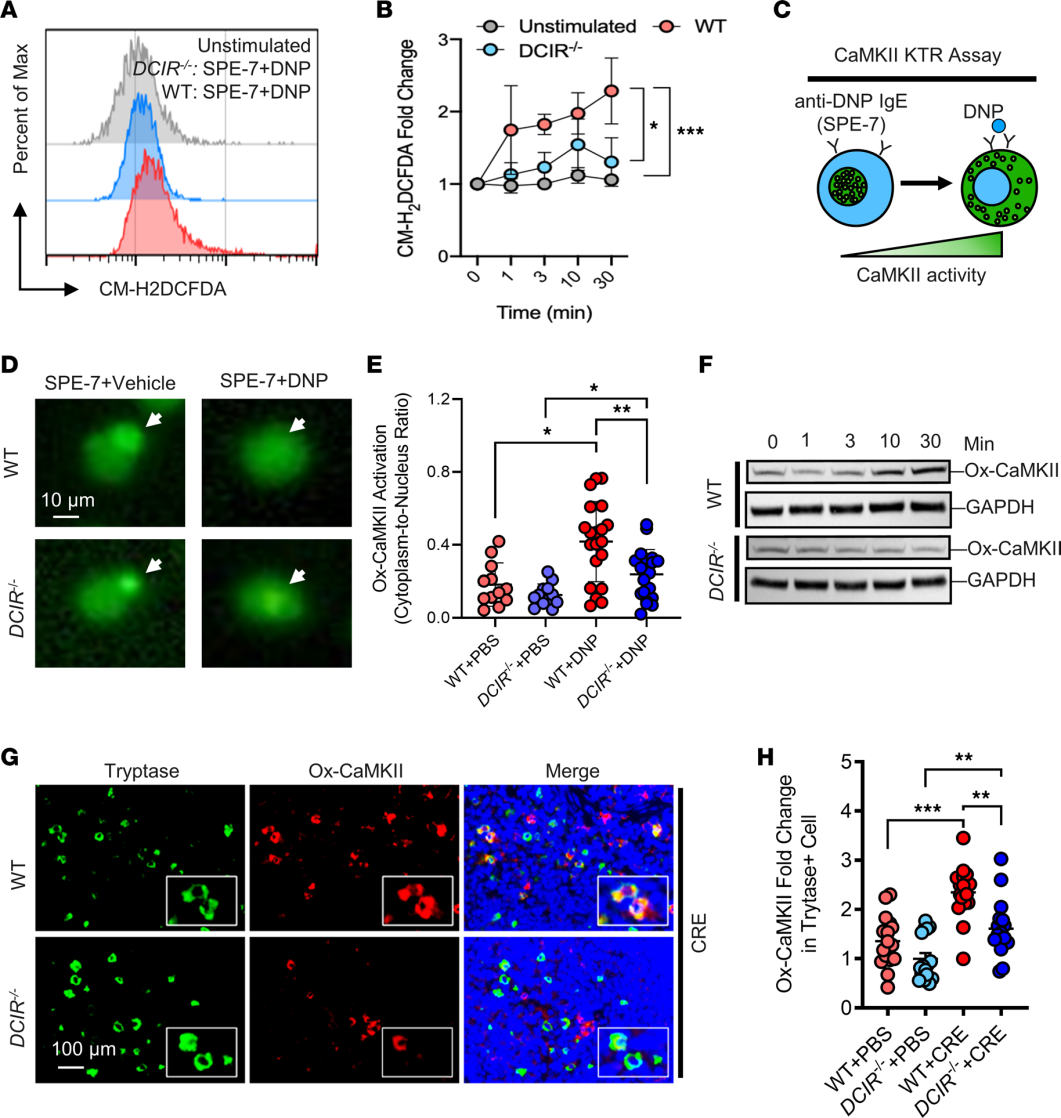
DCIR regulates ROS generation and CaMKII oxidation. (**A**) Flow cytometry analysis of intracellular ROS production with CM-H2DCFDA. (**B**) Quantitative analysis of flow cytometry data (*n* = 3–5). (**C**) Schematic of the CaMKII kinase activity translocation reporter assay (CaMKII-KRT). (**D** and **E**) Representative immunofluorescence images of CaMKII-KTR transfection into WT or *DCIR^–/–^* BMMCs and then treated with or without DNP (white arrows indicate the location of the nucleus) (**D**) and quantification of cytosolic to nuclear KTR signal ratios (*n* = 12–20) (**E**). Scale bar: 10 μm. (**F**) Western blot analysis of ox-CaMKII expression in DNP-treated BMMCs from WT and *DCIR^–/–^* mice. (**G** and **H**) Representative immunofluorescence images of dorsal skin sections (**G**) and quantitative fluorescence analysis (**H**) of ox-CaMKII staining in the skin mast cells of CRE-treated WT and *DCIR^–/–^* mice (*n* = 16). Scale bar: 100 μm. Data represent mean ± SEM of 2 independent experiments. Data were compared by 2-way ANOVA. *P < 0.05, **P < 0.01, ***P < 0.001.

Our previous studies have demonstrated that oxidized CaMKII (ox-CaMKII) regulates mast cell activation (53, 54). These findings raise the possibility that DCIR regulates mast cell activation through controlling ROS generation and oxidative activation of CaMKII. Indeed, our flow cytometry analysis of intracellular ROS production with CM-H2DCFDA showed that ROS expression was reduced in DNP-treated *DCIR^–/–^* BMMCs compared with WT BMMCs (Figure 8, A and B). Next, we tested whether DCIR regulates allergen-induced CaMKII activity by using a CaMKII activity sensor CaMKII-KTR-GFP (Supplemental Figure 7A) (56, 57) following the protocol as illustrated in Figure 8C. Transfection of CaMKII-KTR into BMMCs was confirmed with GFP under the microscope (Supplemental Figure 7B), and CaMKII activity was determined by the cytosol/nuclear distribution of the KTR. As expected, the nuclear KTR was translocated into the cytosol after treatment with DNP in WT BMMC, but the translocation observed was blunted in *DCIR^–/–^* BMMCs, indicating that DCIR may modulate the allergen-induced CaMKII activity (Figure 8, D and E). The same pattern was observed for CaMKII oxidation in mast cells. Western blot analysis demonstrated that DNP treatment induced ox-CaMKII expression in WT BMMCs but not in *DCIR^–/–^* BMMCs (Figure 8F). This finding was further supported by ox-CaMKII expression in AD mouse model. Compared with WT, *DCIR^–/–^* mice showed reduced expression of ox-CaMKII in skin mast cells (Figure 8, G and H). Together, the results indicate that DCIR modulates ROS generation and ox-CaMKII expression that may contribute to mast cell activation.

### ROS-resistant CaMKII protects against allergen-induced mast cell activation.

**Figure 9:**
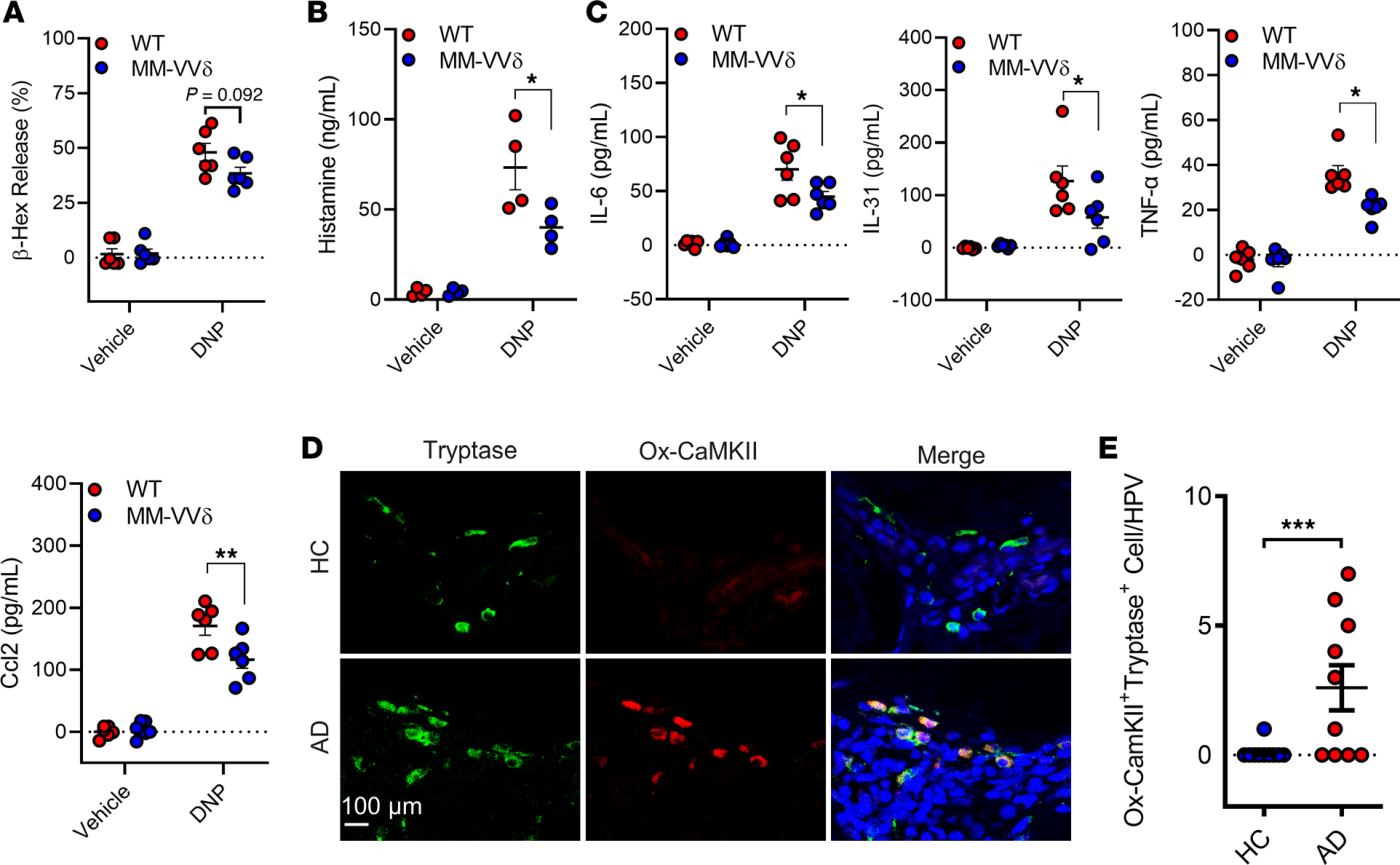
ROS-resistant CaMKII protects against allergen-induced mast cell activation. (**A**–**C**) Analyses of β-hexosaminidase (**A**, *n* = 6), histamine release (**B**, *n* = 4), and cytokines and chemokines (**C**, *n* = 6) in supernatants of DNP-activated BMMCs derived from WT and CaMKII MM-VVδ mice. (**D**) Representative immunofluorescence images of dorsal skin sections and fluorescence analysis of ox-CaMKII staining in the skin mast cells of patients with AD and controls. Scale bar: 100 μm. (**E**) Quantitative analyses for total number of ox-CaMKII^+^ mast cells (*n* = 10). Data represent mean ± SEM of 2 independent experiments. Data were compared using a 2-tailed Student’s *t* test. *P < 0.05, **P < 0.01, ***P < 0.001.

Next, we examined whether ox-CaMKII plays a role in DCIR-regulated, IgE-mediated mast cell activation by using BMMCs from ROS-resistant CaMKII (MM-VVδ) mice. Flow cytometry analysis demonstrated that BMMCs from the ROS-resistant CaMKII (MM-VVδ) mice showed a marked reduction in the levels of β-hexosaminidase after DNP treatment when compared with those from WT mice (Figure 9A). A similar trend was noted for histamine release (Figure 9B). Furthermore, we examined whether ox-CaMKII contributes to mast cell mediator release that was observed in mast cells from *DCIR^–/–^* mice. As expected, IL-6, IL-31, TNF-α, and CCL2 were much lower in supernatants of DNP-treated mast cells from MM-VVδ mice compared with those from WT mice (Figure 9C). Given the significance of ox-CaMKII in mast cell activation, we analyzed ox-CaMKII expression in skin tissues from patients with AD. As expected, skin tissues from patients with AD showed an increased expression of ox-CaMKII in mast cells compared with those from healthy controls according to the total number of *ox-CaMKII^+^* mast cells (Figure 9, D and E). These findings suggest that ox-CaMKII may regulate the allergen-induced mast cell activation and inflammatory mediator release in patients with AD.

## Discussion

There is increasing evidence that exposure to allergens is one of the major risk factors for the development of AD; provoking skin inflammation in sensitized patients with AD through IgE-dependent and cell-mediated immune responses has shown these results (3, 58). S.c. allergen immunotherapy has been suggested to be an effective therapeutic approach for AD (59, 60), implying that allergen plays a pathogenic role in the development of AD. To better understand the role of cutaneous exposure to aeroallergens in AD, several mouse models of allergen-induced AD-like skin inflammation have been established and recognized as powerful approaches to analyze the allergen-induced pathophysiology of AD and elucidate the underlying mechanisms (43–45, 61–63). Of these, the OVA-induced AD mouse model has been well established (43–45); we therefore used OVA as a positive control to establish a new AD-like mouse model by using cockroach allergen. As expected, cockroach allergen–induced mice showed thickening of the epidermal and dermal layers and infiltration of inflammatory cells. These mice displayed increased skin infiltration of Th2, ILC2, and mast cells and showed elevated Th2 and inflammatory cytokine expression that have been well documented for human AD (3, 4, 43, 49), suggesting that our newly generated mouse model is a useful model to study the allergen-induced pathogenic processes and its underlying mechanisms.

In this study, we specifically focused on mast cells and investigated the role of mast cells in mediating allergen-induced AD-like skin inflammation. Skin mast cells are strategically positioned and equipped to respond to environmental allergens and serve as a link between innate and adaptive immunity (43). Studies have demonstrated increased numbers of mast cells in the skin lesions of patients with AD (32, 33) and mouse models (34, 35). Active mast cells can release numerous biologically active mediators to alter the activation and functions of the surrounding immune cells and subsequently lead to skin inflammation and dermatitis (64–66). Our current study selected *Kit^W-sh/W-sh^* mice that are widely used to analyze the functions of mast cells in vivo (67) to determine whether mast cells are required in the cockroach allergen–induced mouse model of AD. Our findings support previous findings that mast cells are increased not only in the lesional skins of patients with AD, but also in the skin tissues of our AD mouse model. Importantly, we provided additional evidence that mast cells are required in the development of skin allergic inflammation by using mast cell–deficient *Kit^W-sh/W-sh^* mice. However, due to the cKit expression on other cells (e.g., ILC2s), we recognize that *Kit^W-sh/W-sh^* mice not only exhibit a profound mast cell deficiency, but also a variety of other phenotypic abnormalities (68, 69). Thus, our future study will further characterize the role of mast cells in a cockroach allergen–induced mouse model of AD by using different mast cell–deficient lineages of mice.

To further explore how mast cells contribute to the skin allergic inflammation, we focused on DCIR, one of the CLRs. CLRs are expressed in different types of immune cells (e.g., DC and macrophages) and play a role in facilitating the uptake of antigens and regulating downstream immune responses (70–72). However, there are currently few studies on CLR expression within mast cells and their function (73, 74). DCIR as one of the major CLRs expressed on DCs has a carbohydrate recognition domain in its extracellular portion (19, 20) and plays a role in carbohydrate recognition and ITIM signaling–mediated immune regulation (22, 71). Intriguingly, studies have indicated that DCIR is required for the development of autoimmune diseases (25) and is essential for the modulation of immunity to tuberculosis (24). We have recently made a finding that DCIR is highly expressed in basophils and mediates cockroach allergen uptake (13). These findings led us to hypothesize that DCIR is also expressed in mast cells and participates in mast cell–mediated allergen-induced skin inflammation. Indeed, we have demonstrated that DCIR is highly expressed in skin mast cells of patients with AD and in the mouse model. Importantly, our in vitro analysis suggests that DCIR is involved in allergen binding and uptake by mast cells, as evident by the fact that the uptake of FITC-CRE was blocked by DCIR neutralizing antibody. However, no data were provided to directly see the cockroach allergen skin penetration and binding on mast cells in vivo, which was limited by the complexity of CRE and availability of the techniques optimized to track the allergen skin penetration and binding in the mouse model of AD. Given the fact that both direct and indirect mechanisms have been recognized for the mast cell–pathogen interactions (75), it is likely that DCIR is a previously unrecognized CLR expressed on mast cells that modulates allergen-induced mast cell activation in vivo through direct mechanisms, which would be of interest for future elucidation.

Given the expression of DCIR in mast cells and its pivotal role in allergen binding and uptake, we provided further evidence that DCIR plays an important role in the pathogenesis of AD. First of all, by using our established AD mouse model, *DCIR^–/–^* mice revealed attenuation of allergen-induced skin inflammation, as evident by reduced EASI score, epidermal thickness, and skin expression of Th2 and skin epithelial cell–derived cytokines. Next, to test whether the attenuated skin inflammation observed in *DCIR^–/–^* mice is due to the DCIR deficiency specifically in mast cells, we generated “mast cell knock-in mice” by restoring the mast cell deficiency in *Kit^W-sh/W-sh^* mice by adoptively transferring WT and *DCIR^–/–^* mast cells. Of interest, mice with *DCIR^–/–^* mast cell knock-in showed attenuated skin inflammation as compared with those with WT mast cell knock-in, suggesting that DCIR in mast cells is critical in mediating cockroach allergen–induced AD-like inflammation. Next, we explored whether DCIR is involved in regulating allergen-induced mast cell activation and inflammatory mediator release. It is well established that IgE plays a critical role in the pathogenesis of AD, and targeting IgE has been considered as one of the effective treatments for patients with AD (76). This study illustrated increased levels of allergen-specific IgE and higher numbers of active mast cells; the classical IgE/FcεRI/mast cell pathway is still crucial for cockroach allergen–induced AD-like inflammation. Recent studies have also suggested several IgE-independent mechanisms underlying the allergen-induced mast cell activation (e.g., IgG/FcγRs, C3a and C5a, TLR2, drugs/receptors [MRGPRX2]) (42, 77). Indeed, we used the FcεRI cross-linking with anti–DNP-IgE to activate mast cells, a well-established in vitro model (78), and found that *DCIR^–/–^* mast cells had a significant reduction in the expression of CD107a (LAMP-1) and the release of β-hexosaminidase and histamine from mast cells. Thus, although no difference was noted for the IgE receptor FcεRI for WT and *DCIR^–/–^* mast cells, our in vitro analysis demonstrated that *DCIR^–/–^* mast cells had a significant reduction in IgE-mediated mast cell activation. The findings were further supported by our in vivo PCA assay, an animal model for inflammatory reactions in Type I allergy, indicating that *DCIR^–/–^* mast cells had a significant reduction in IgE-mediated cutaneous anaphylaxis. Furthermore, we characterized the role of IgE in cockroach allergen–induced AD-like inflammation by using IgE-KO mice. Interestingly, all the phenotypic changes observed in IgE-KO mice appeared comparable with WT mice, implying that other factors beyond IgE-mediated pathway play roles in cockroach allergen–induced mast cell activation and AD. Together, these data suggest that DCIR on mast cells may represent an IgE-independent pathway but participate in regulating IgE-mediated allergen-induced mast cell activation and skin inflammation in AD. The detailed mechanisms underlying the cross-talk between DCIR and IgE signaling pathways in mast cell activation are still unclear, warranting further, in-depth investigations.

In the present study, we have also explored the underlying mechanisms by which DCIR regulates allergen-induced mast cell activation. The CaMKII is a serine/threonine-specific protein kinase that can be activated by ROS at methionines 281/282 in the regulatory domain, leading to a persistently oxidative activation of CaMKII (79–82).We have recently demonstrated the significance of ox-CaMKII in regulating mast cell activation (53, 54) and epithelial cell autophagy/mitophagy (83). These findings led us to investigate whether DCIR regulates mast cell activation through modulating ROS generation an oxidative activation of CaMKII in AD. Indeed, we found that allergen-sensitized and -challenged *DCIR^–/–^* mast cells showed significantly reduced intracellular ROS production, suggesting that ROS may be a central player linking DCIR and IgE/FcεRI pathways with mast cell activation. Furthermore, we used a unique CaMKII activity sensor CaMKII-KTR and found that DCIR in mast cells is essential in modulating allergen-induced CaMKII activity, as evident by a clear translocation of CaMKII-KTR from nuclear to cytosol after treatment with DNP. The CaMKII-KTR has been used to detect CaMKII activity in a variety of cell types (56, 57). The results were further supported by the DCIR-regulated allergen-induced CaMKII oxidation. Collectively, these findings suggest a potential mechanism that DCIR regulates allergen-induced mast cell activation through controlling allergen-induced mast cell ROS generation and oxidation of CaMKII.

While the present study mainly focused on the innate immunity, with an aim being to elucidate the role of mast cells in response to cockroach allergen, it remains unclear about the complicated effector mechanisms underlying the mast cell–mediated adaptive immunity. It has been suggested that mast cells not only release mediators (e.g., IL-4, IL-13, CCL2, histamine, tryptase, LTC4, PGD2, and PGE2) during degranulation in response to environmental allergens, but also interact with resident or recruited immune effector cells, leading to the development of skin inflammation (75). We have found increased skin infiltration of Th2, ILC2, and mast cells and elevated Th2 and inflammatory cytokine expression in the cockroach allergen–induced AD-like inflammation, and *DCIR^–/–^* mast cells secreted less IL-6, IL-31, CCL2, and TNF-α relative to WT mast cells. Thus, it is possible that mast cells may interact with these recruited cells (e.g., ILC2, Th2) through those released mediators that control cockroach allergen–induced AD-like inflammation.

Taken together, we established a new AD-like mouse model by using cockroach allergen and provided evidence that mast cells are increased in the lesional skin of patients with AD and mouse model and are also essential in cockroach allergen–induced skin allergic inflammation. Further in vitro analyses identified DCIR in mast cells that plays an important role in allergen binding and uptake and in modulating IgE-mediated mast cell activation and inflammatory mediator release. In addition, we demonstrated that DCIR in mast cells is IgE independent but involved in regulating IgE-mediated allergen-induced mast cell activation. Mechanistic studies suggest that DCIR regulates allergen-induced IgE-mediated mast cell activation through controlling allergen-induced mast cell ROS generation and ox-CaMKII expression, leading to mast cell degranulation and overproduction of inflammatory mediators that trigger skin AD-like inflammation in AD. Our results suggest an important functional axis of DCIR/ROS/CaMKII in mast cells that may represent an IgE-independent pathway, but the axis participates in regulating IgE-mediated allergen-induced mast cell activation and skin inflammation in AD, thereby highlighting the therapeutic potential for patients with AD.

## Methods

### Human AD skin.

Patients with AD were enrolled at Pediatric Dermatology, Children’s Hospital of Chongqing Medical University, who fulfilled the diagnostic criteria of Hanifin and Rajka (84). Children with systemic diseases, acute infection, and autoimmune diseases were excluded from the study. The disease severity was evaluated based on the Scoring of Atopic Dermatitis (SCORAD) (85). The clinical and demographic data of human subjects including patients and healthy controls were provided in Supplemental Tables 1 and 2. AD skin tissues were collected from patients with AD by punch biopsy at lesional skin on the arms, legs, or trunks at the Children’s Hospital of Chongqing Medical University. Normal skin tissues were obtained from plastic surgery. Skin tissues were formalin-fixed, paraffin-embedded and sectioned (4 μm) for H&E and TB staining to measure the epidermal thickness and the numbers of mast cells.

### Mice.

C57BL/6, *Kit^W-sh/W-sh^*, and BALB/C mice were purchased from The Jackson Laboratory. *DCIR^–/–^* mice were provided by Bernd Lepenies at The University of Veterinary Medicine Hannover (Hannover, Germany) and were bred in-house. CaMKII MM-VVδ mice were provided by Mark Anderson at Johns Hopkins University (53). IgE-KO mice were provided by Hans C. Oettgen at the Boston Children’s Hospital in Harvard Medical School (Boston, Massachusetts, USA) (86). All experiments were conducted using age- and sex-matched 6- to 12-week-old male and female mice. All mouse strains were bred and maintained under the same specific pathogen–free conditions at an American Association for the Accreditation of Laboratory Animal Care–accredited (AAALAC-accredited) animal facility at Johns Hopkins University and housed according to procedures described in the *Guide for the Care and Use of Laboratory Animals* (National Academies Press, 2011).

### Establishment of cockroach allergen–induced AD model.

A mouse AD model was generated as shown in Figure 1A, which was modified from previous reports (43–45). The hair on the back skin of anesthetized mice was clipped by using electric clippers. Residual hair was depilated by using a Nair hair removal cream. Antigen challenge was performed by using one square centimeter of gauze containing 100 μg of CRE (Greer Laboratories) or OVA (MilliporeSigma) in PBS pipetted onto a 1 cm square sterile gauze pad and placed on the dorsal shaved skin. Control mice received PBS alone. The patched skin area was sealed with a Tegaderm Transparent dressing (3M HealthCare) using bandages. These procedures were repeated twice a week for 3 weeks with 1-week interval. The severity of skin inflammation (e.g., erythema/hemorrhage, eruption, scarring/dryness) was evaluated using EASI score on day 2 after the patch removal as 0 (no symptom), 1 (mild), 2 (intermediate), or 3 (severe). The total score of skin lesions was designated as the sum of individual scores. After final evaluation, mice were sacrificed, and serum, lesion, and nonlesion skin tissues were collected.

### Histology and immunofluorescence.

Specimens from the dorsal skin were collected and then fixed in 4% formalin solution for 24 hours before embedding in paraffin and sectioned at 4 μm for histological staining. The fixed sections were deparaffinized with xylene, rehydrated with ethanol, blocked for endogenous peroxidase with methanol, and then stained with H&E and TB, respectively. Epidermal and dermal thickness and skin mast cells were evaluated by light microscopy. For immunofluorescence staining, 4 μm sections were used and stained as previously reported (87). Briefly, the sections were incubated for 15 minutes at 95°C, washed with antigen retrieval solution (Dako), and then blocked with 20% FBS in Tris-buffered saline (TBST; 0.1% Tween 20) for 1 hour at RT. The sections were then incubated with the primary antibodies against mouse Tryptase (AF1937, R&D system), DCIR (MAB2617, R&D), or mouse IgG1 overnight at 4°C. The sections were washed with TBST and then incubated with fluorescent dye–conjugated secondary antibodies for 30 minutes at RT. Detailed information about the antibodies is provided in Supplemental Table 3. Nuclei were counterstained with DAPI (Invitrogen). The sections were observed by a NIKON ECLIPSE Ti-U microscope equipped with DS-Fi2 camera (NIKON). The fluorescent-positive cells were evaluated in 4 different high-power fields from each skin section using ImageJ v1.50e (NIH). Four to 6 skin sections from each sample were used for analysis.

### FACS analysis.

Flow cytometry was performed as previously described (48). Briefly, 10 mm skin punch biopsies were collected, minced, and enzymatically digested in 3 mL RPMI containing 100 μg/mL DNaseI (Sigma-Aldrich) and 1.67 Wunsch U/mL Liberase TL (Roche) for 1 hour at 37°C and shaken at 140 rpm. Single-cell suspensions were obtained after filtering the digested samples through a 40 μm cell filter using a 3 mL syringe plunger, and cells were then washed in RPMI. The single-cell suspension was incubated with TruStain fcX (BioLegend) to block Fc receptor binding and resuspended to label with mAbs against cell surface markers. Markers used for flow cytometry analysis and detailed methods are provided in the Supplemental Methods and in Supplemental Table 3. Cell acquisition was performed on the BD LSRFortessa (BD Biosciences) and FACSCalibur flow cytometer (BD Biosciences), and data were analyzed using FlowJo software (v10) (Tree Star Inc.). Gating of single cells was done using FSC/W and SSC/W, and exclusion of dead cells was accomplished with the LIVE/DEAD Fixable Far-Red Dead Cell Stain (Thermo Fisher Scientific).

### Quantitative PCR.

Skin tissues were homogenized using a Percellys Homogenizer (Bertin Technologies) with 1.0 mm zirconium oxide beads, and total RNA was extracted from skin tissues using the Monarch Total RNA isolation kit (New England Biolabs) according to the manufacturer’s instructions. cDNA was synthesized by using High-Capacity RNA-to-cDNA Kit (Applied Biosystems). Quantitative PCR (qPCR) was performed using SYBR Green PCR Master Mix (Applied Biosystems) using a 7300 Real-Time PCR System (Applied Biosystems). Gene relative expression was calculated using the 2^−ΔΔCt^ method as described by Livak and Schmittgen (88). The mRNA levels were normalized to the internal gene (*β-actin*). Primer sequences are provided in Supplemental Table 4.

### ELISA.

Levels of cytokines in serum were quantified by using the Ready-Set-Go! ELISA sets (Thermo Fisher Scientific). Allergen-specific IgE and IgG1 serum levels were analyzed by ELISA as previously described (89).

### Western blotting.

For Western blotting analysis, an equal amount of total protein (20–50 μg) was loaded onto a 12% Tris-Glycine Gel in NuPAGE MES SDS Running Buffer (Thermo Fisher Scientific) and then transferred using the iBlot2 NC Stack System (Thermo Fisher Scientific). The membranes were blocked in 5% nonfat milk in TBST for 1 hour at RT and probed with primary antibodies overnight at 4°C. Species-appropriate secondary antibodies conjugated to IRDye 680RD or IRdye 800CW (LI-COR Biosciences) were used according to the manufacturer’s instructions. Band intensities were quantified by ImageJ software, and quantification on each band was normalized to β-actin. Antibody usage is provided in Supplemental Table 5.

### Solid-phase binding assay.

Enzyme immunoassay/radioimmunoassay (EIA/RIA) 96-well flat-bottom plates (Costar) were coated in duplicate with 10 μg/mL of CRE, mannan-BSA, or BSA in PBS overnight at 4^o^C. The plate was blocked with 1% BSA in TBST for 1 hour at RT. Various concentrations of purified recombinant human DCIR-6xHis (Sino Biological) dissolved in blocking buffer with 100 μg/mL of CaCl_2_ were incubated for 1 hour at RT. The plate was washed and incubated with anti–6xHis horseradish peroxidase–conjugated IgG (MAB050H, R&D Systems; Supplemental Table 5). The absorbance was recorded at 450 nm using an iMark Microplate Absorbance Reader (Bio-Rad).

### Generation of BMMCs.

Mouse BM-derived mast cells (BMMCs) from C57BL/6J and *DCIR^–/–^* mice were cultured as previously described (53–55). Briefly, BM cells were cultured at a starting density of 1 × 10^6^ cell/mL in the presence of 10 μg/mL of mouse recombinant IL-3 (BioLegend) for 5 weeks as previously described (53). Mast cell phenotype was confirmed by TB staining and flow cytometry analysis with antibodies specific for cKit (1:100, 2B8, eBiosciences) and FcεRI (1:100, MAR-1, eBiosciences).

### Mast cells reconstitution in Kit^W-sh/W-sh^ mice.

BMMCs were cultured from WT and *DCIR^–/–^* mice and adoptively transferred into *Kit^W-sh/W-sh^* mice. A total of 1 × 10^7^ BMMCs in 200 μL PBS were adoptively transferred into *Kit^W-sh/W-sh^* mice via tail vein to systemically reconstitute mast cells. In the meantime, *Kit^W-sh/W-sh^* mice received PBS only as the control. Skin biopsies were taken at 4 weeks after mast cell transferring and then stained with TB to confirm the presence of mast cells. Mast cell–reconstituted mice were used to generate a CRE-induced AD model by using the same protocol as described above.

### BMMC activation.

For mast cell activation, BMMCs were sensitized with 0.5 μg/mL anti–DNP-IgE (clone SPE-7; Sigma-Aldrich) overnight at 37°C at a density of 1 × 10^6^ cell/mL. Cells were washed, resuspended in Tyrode’s buffer, and challenged with 100 ng/mL of DNP-HSA (Sigma-Aldrich) and 2.5 μg/mL ionomycin (Sigma-Aldrich) for 30 minutes at 37°C. Mast cell activation was assessed by the analyses of β-hexosaminidase, histamine release, and CD107a (LAMP-1). Briefly, β-hexosaminidase was expressed as a percentage of β-hexosaminidase calculated from the total β-hexosaminidase in 0.5% Triton X-100 lysed BMMCs as previously described (53). Histamine release was measured after 30 minutes by taking the top 0.05 mL of culture supernatant, diluting in 1 mL acid solution for overnight protein precipitation, and assaying using automated fluorimetry as previously described (90). Expression of CD107a (LAMP-1) as one of the mast cell activation markers was detected by flow cytometry analysis. Intracellular ROS were detected using the oxidative sensitive fluorescent dyes CM-H_2_DCFDA (Thermo Fisher Scientific) as previously reported (87).

### CaMKII KTR assay.

To analyze CaMKII activity, BMMCs were transfected with a CaMKII activity sensor CaMKII-KTR-GFP using Lipofectamine 3000 (Thermo Fisher Scientific). The CaMKII-KTR was provided by Mark Anderson at Johns Hopkins University. Its construction and validation have been previously reported (56, 57). After transfection for 24 hours, BMMCs were sensitized and challenged, and they were imaged using a Nikon ETi fluorescent microscope equipped with a sCMOS PCO.edge monochromatic camera. Images were analyzed by using Nikon NIS-Elements D (version 5.21.01). The cytosolic to nuclear KTR-GFP signal ratios were calculated using the AUC fluorescent signals measured from the nuclei and cytoplasm of individual cells.

### Multiplex cytokine screening.

For multiplex cytokine screening, BMMCs were sensitized and challenged by using the same methods as described for mast cell activation. Supernatants were used to test cytokine release with the MSD U-PLEX Biomarker Group 1 (Mouse) Multiplex Assays as per manufacturer’s instructions. Chemiluminescent images of array blots were captured using a MyECL imager (Thermo Fisher Scientific), and spot intensity was analyzed using Fiji/ImageJ by background correction of images and integrated density measurements of circular selections.

### Passive cutaneous anaphylaxis assay.

For PCA assay, WT and *DCIR^–/–^* mice were sensitized by intradermal injection of 100 ng anti–DNP-IgE in 20 μL PBS in the ear. After 48 hours, the mice were injected via tail vein with 200 μg DNP-HSA in 200 μL 4% Evans blue dye in saline for 30 minutes. For CRE-induced cutaneous anaphylaxis assay, 25 μg of CRE in 20 μL of PBS were injected through intradermal injection into the right pinnae of WT and *DCIR^–/–^* mice. Left pinnae were injected with 20 μL of PBS as a control. After 30 minutes, the mice were injected via tail veil with 200 μL 4% Evans blue dye in saline for 30 minutes. Finally, ear tissues were excised, and Evans blue dye was extracted with formamide at 55°C overnight. Absorbance was measured at 620 nm, and data are expressed as Evans blue in ng/mg tissue.

### Statistics.

All results were expressed as means ± SEM. Statistical significance was determined using a 2-tailed Student’s *t* test for 2 data sets and 1-way ANOVA (*P* value adjusted for multiple comparisons by Tukey’s test). Statistical analyses were performed using the Graph Pad Prism software program (GraphPad Prism 8). *P* ≤ 0.05 was considered statistically significant.

### Study approval.

The animal care and experiments were performed in compliance with the institutional and US NIH guidelines and were reviewed and approved by the Johns Hopkins University Animal Care and Use Committee. AD skin tissues were collected from patients with AD by punch biopsy at lesional skin on the arms, legs, or trunks at the Children’s Hospital of Chongqing Medical University. The study protocol was approved by the Children’s Hospital of Chongqing Medical University Institutional Board (file no. 2018.73). All the patients and healthy donors consented through written and informed agreement for inclusion in the study.

## Author contributions

XL, JC, HY, XH, MPA, YS, XZ, WT, and YK performed experiments and analyzed data. XL, JC, HW, and PG wrote the manuscript. TK, MW, NA, HW, and PG designed and supervised the study, and revised the manuscript. All authors read and approved the submitted manuscript.

## Supplementary Material

Supplemental data

## Version 1 02/03/2022

In-Press Preview

## Version 2 03/08/2022

Electronic publication
