# Supplementary material for: Dendritic cell immunoreceptor drives atopic dermatitis by modulating oxidized CaMKII-involved mast cell activation
Source: JCI Insight. 2022 Mar 8;7(5):e152559. doi: 10.1172/jci.insight.152559 (PMC8983143; doi:10.1172/jci.insight.152559)

Supplementary Materials

Dendritic Cell Immunoreceptor Drives Atopic Dermatitis by Modulating Oxidized CaMKII-Involved Mast Cell Activation

Xiaoyan Luo[1,2†], Jingsi Chen[1,2†], Huan Yang[1,2†], Xinyue Hu[1,3], Martin P Alphonse[4], Yingchun Shen[1], Yuko Kawakami[5], Xiaoying Zhou[2], Wei Tu[1], Toshiaki Kawakam[5, 6], Mei Wan[7], Nathan Archer[4], Hua Wang[2]*, Peisong Gao[1]*

[1]Division of Allergy and Clinical Immunology, Johns Hopkins University School of Medicine; Baltimore, MD 21224, USA

[2]Pediatric Dermatology, Children's Hospital, Chongqing Medical University; Chongqing 400014, China

[3]Department of Respiratory Medicine, Xiangya Hospital, Central South University; Changsha, Hunan 410008, China

[4]Department of Dermatology, Johns Hopkins University School of Medicine; Baltimore, MD 21231, USA

[5]Division of Cell Biology, La Jolla Institute for Allergy and Immunology; La Jolla, CA 92037, USA

[6]Department of Dermatology, School of Medicine, University of California San Diego; La Jolla, CA 92093, USA

[7]Department of Orthopaedic Surgery, Johns Hopkins University School of Medicine, Baltimore, Maryland, USA

[†]These authors contributed equally to this work

METHODS

Knockdown of human DCIR

LAD2, an immortalized human mast cell line (Gift from NIH), was cultured in StemPro-34 serum-free media (Gibco) supplemented with penicillin (50 IU/ml), streptomycin (50 µg/ml), L-glutamine (2 mM), and recombinant human stem cell factor (100 ng/ml). DCIR knockdown was performed by using a pre-designed Mission siRNA pair. The siRNA transfection was carried out using Lipofectamine™ RNAiMAX (ThermoFisher) according to the manufacturer's instructions.

FACS analysis

Flow cytometry was performed as described earlier (1). In brief, 10-mm skin punch biopsies were collected, minced and enzymatically digested in 3 mL RPMI containing 100 µg/mL DNaseI (Sigma-Aldrich) and 1.67 Wunsch U/ml Liberase TL (Roche) for 1 hour at 37°C and shaken at 140 rpm. Single-cell suspensions were obtained after filtering the digested samples through a 40-µm cell filter using a 3 mL syringe plunger and cells were then washed in RPMI. The single-cell suspension was incubated with TruStain fcX (Biolegend) to block Fc receptor binding and resuspended to label with mAbs against cell surface markers. The cell surface markers included CD45, Lineage marker, cKit (CD117), KLRG1, CD127, CD11b, CD3, CD4, CD8, gdTCR, and FcεRI, in Hanks Balanced Salt Solution (HBSS) with 2% Calf Serum and 5mM Hepes along with Brilliant Stain Buffer (BD Biosciences). The cells were washed with PBS and stained for viability (Zombie Aqua Fixable Viability Kit – BioLegend). The surface labeled cells were fixed in the BD Cytofix/ Cytoperm Buffer kit (BD Biosciences). The cells were further labeled for intracellular cytokines, including IFNg, TNF, IL-17, and IL-4. Respective IgG isotype control for Lineage marker controls were performed in the experiment. The mAb-labeled cells were then washed in intracellular staining buffer and resuspended in Stabilizing Fixative (BD Biosciences). Cell acquisition was performed on the BD LSRFortessa flow cytometer (BD Biosciences)

and data were analyzed using FlowJo software (v10). For immunophenotyping of ILC2, cells were first gated on live cells, singlets and $CD45^+$, Lin^- populations, and further gated on $KLRG1^+CD127^+CD25^+$ cells. For identification of Th1 ($IFNg^+$), Th2 ($IL\text{-}4^+$) and Th17 ($IL\text{-}17^+$) population, cells were over-laid with expression of $CD4^+$ T-cells ($CD45^+CD3^+CD4^+CD8^-gdTCR^-$), $CD8^+$ T-cells ($CD45^+CD3^+CD8^+$), and γδ-T-cells ($CD45^+CD3^+CD8^-gdTCR^+$).

ELISA

Levels of cytokines in serum were quantified by using the Ready-Set-Go! ELISA sets (ThermoFisher) (2). Cockroach allergen-specific IgE and IgG1 serum levels were analyzed by ELISA as previously described (3). Briefly, 96-well flat-bottom plate (Costar, Columbia, MD, USA) was coated with allergen (10μg/ml) in PBS overnight at 4°C and blocked with 5% BSA in TBST for 1 h at room temperature. Serum was diluted at 1:1000 in PBS for IgG1 detection or treated with rec-Protein G-Sepharose 4B (ThermoFisher) for IgE detection. Serum samples were added and incubated overnight at 4°C. Serum IgE/IgG1 antibody was then measured using a biotinylated anti-mouse IgE/IgG1 (Biolegend), followed by an HRP-conjugated antibody to mouse IgE/IgG1. Absorption at 450 nm was measured with an iMark Microplate Absorbance Reader (BioRad).

FIGURE LEGENDS

Fig, S1. Gating strategy for cell populations from skin tissues of AD mouse model. (**A**). Representative flow plot gating strategy for T-cells, including CD8$^+$ T-cells CD4$^+$ T-cells (CD45$^+$CD3$^+$CD4$^+$CD8$^-$gdTCR$^-$), CD8$^+$ T-cells (CD45$^+$CD3$^+$CD8$^+$), γδ-T-cells (CD45$^+$CD3$^+$CD8$^-$gdTCR$^+$), Th1 (IFNγ$^+$), Th2 (IL-2$^+$), Th17 (IL-17$^+$) T-cells, and mast cells (CD45$^+$CD3$^-$FcεRI$^+$cKit$^+$), respectively. (**B**) Representative flow plots for the ILC2 population (CD45$^+$Lin$^-$KLRG1$^+$CD127$^+$CD25$^+$).

Fig. S2: **Increased mast cells and DCIR expression in the lesional skins of AD patients.** (**A-B**) Representative hematoxylin and eosin (H&E, **A**) and Toluidine blue (**B**) staining of skin tissue sections (arrow, mast cell) of AD patients (AD, n=15) and healthy controls (HC, n=15). (**C-D**) Quantification of epidermal thickness (**C**, μm) in (**A**) and positive staining (**D**) in (**B**) for Toluidine blue. n=10/group. (**E**) RT-PCR analysis of DCIR expression in the skin tissues of AD patients and controls (n=8). Data were compared using a two-tailed Student's t-test. ***P <0.001.

Fig. S3. Cockroach allergen-induced skin inflammation is independent of IgE. (**A**) Serum levels of specific IgE and IgG1 to PBS or CRE of WT and IgE knockout mice (IgE KO) mice exposed to PBS or CRE. (**B**) Representative hematoxylin and eosin (H&E) staining and epidermal thickness (μm) of skin tissues of WT and IgE knockout mice (IgE KO) mice exposed to PBS or CRE. (**C**) Quantitative RT-PCR analysis of IL-4 and IL-13 expression in the skin tissues of PBS or CRE-treated mice. (**D**) Serum levels of IL-4 as assessed by ELISA. n=2-7. Data represent mean ± SEM. Data were compared by using 2-way ANOVA. **P <0.01, ***P <0.001.

Fig. S4. DCIR expression in skin mast cells of the AD mouse model. (**A**) Representative immunofluorescence images of dorsal skin sections and fluorescence analysis of DCIR staining in the epidermis of PBS and CRE-treated WT or $Kit^{W\text{-}sh/W\text{-}sh}$ mice. (**B**) Quantification analysis of DCIR$^+$tryptase$^+$ cells in the lesion skin. n=8/group (**C**) qRT-PCR analysis of DCIR expression in the skin

tissues. n=3/group. Data represent mean ± SEM. Comparisons were made by using 2-way ANOVA. ***P* <0.01, ****P* <0.001.

Fig. S5. DCIR regulates OVA-induced mast cell activation. (**A**) DCIR knockdown by siRNA was confirmed by RT-PCR. (**B-D**) LAD2 with or without DCIR knockdown were sensitized with 1 ug/mL anti-OVA IgE (E-C1) for 16 hours and then stimulated with 10 ug/mL OVA for 30 minutes in Tyrode's buffer. Mast cell activation was assessed by measuring the expression of LAMP-1 (**B**) by flow cytometry, and β-hexosaminidase (**C**) and cytokine levels (**D**) in supernatants by ELISA. n=3-6/group. Data represent mean ± SEM. Data were compared using a two-tailed Student's t-test. **P* <0.05, ****P* <0.001.

Fig. S6. **DCIR regulates IgE-mediated allergic reactions.** (**A**) Scheme of experimental protocol for IgE-mediated anaphylaxis. (**A**) Representative images of Evans blue-stained extravasation into ear skin of CRE-treated WT and DCIR$^{-/-}$ mice and quantification of the extravasation of Evans blue leakage into the skin. n=6/group. (**B**) Representative Toluidine blue staining and quantification of cells with positive staining for Toluidine blue of skin tissue sections of CRE-treated WT and DCIR$^{-/-}$ mice. n=6/group. Data represent mean ± SEM. Comparisons were made using a two-tailed Student's t-test of PBS or CRE-treated WT vs. DCIR$^{-/-}$ mice. **P* <0.05, ****P* <0.001.

Fig. S7. CaMKII kinase activity translocation reporter (CaMKII-KTR). (**A**) Schematic of the CaMKII-KTR. The N-terminus of the KTR is a well-characterized CaMKII-interacting domain from HDAC4 (AA582-62451), followed by a nuclear localization signal (NLS) and a nuclear exporting signal (NES). The high affinity CaMKII substrate consensus sequence (LXRXXSV) was built into both the NLS and NES. The C-terminus of the CaMKII-KTR is an enhanced green fluorescent protein (EGFP). (**B**) The KTR shuttles between the nucleus and cytosol. Phosphorylation by CaMKII decreases the strength of the NLS while increases the strength of the NES, resulting in a net translocation of the KTR into the cytosol. The ratio between the cytosolic and nuclear signals of the KTR corresponds to the

overall activity of CaMKII inside the cells.

Table S1. **Clinical and demographic data of AD patients and healthy control subjects**

Group	n	Age (y)	Sex (Male: Female)	SCORAD	Serum IgE (IU/ml)	Personal or Family History of Allergic Diseases
AD	15	6.8±2.8	9:6	52.9±16.1	426.5±289.8	9/15
Healthy Control	15	6.2±3.2	10:5	_	_	2/15

TABLE S2. Raw data of epidermal thickness and Mast cell number

Group	*Sex*	*Age (y)*	*SCORAD*	*Serum IgE (IU/ml)*	*Biopsy site*	*Epidermal thickness (HE, μm)*	*Num. of MCs (TB, 40X)*
HC							
1	M	8y1m	33.9	3.41	Right leg	116.97	12.5
2	M	3y4m	31.6	-	Left leg	277.31	9.5
3	F	3y11m	28.8	118.6	Trunk	263.14	9
4	M	9y1m	64.4	259	Left arm	158.31	10.6
5	M	4y11m	50.8	58	Left thigh	292.39	9.4
6	F	4y	35.1	679	Right leg	181.89	11.2
7	M	10y	53.2	487	Left thigh	209.55	8.6
8	F	10y6m	72.8	794	Right leg	162.8	11.2
9	F	3y2m	41.2	768	Right forearm	188.8	9.8
10	M	4y4m	58.6	401	Left thigh	107.78	9
11	M	9y4m	67.7	647	Left thigh	157	5.8
12	M	9y11m	60.5	686	Right leg	81.8	5
13	F	5y11m	83.3	314	Left elbow	86.72	11.6
14	F	5y3m	56.8	61	Left thigh	103	7
15	M	9y10m	55	695	Abdomen	91.88	5.6
AD							
1	M	3y6m	-	-	Abdomen	48.67	2
2	M	3y1m	-	-	Abdomen	44.28	3
3	M	1y7m	-	-	Abdomen	42.47	3.5
4	M	5y	-	-	Abdomen	62.65	4.5
5	M	6y	-	-	Abdomen	60.48	6
6	M	5y	-	-	Abdomen	54	1.5
7	F	4y7m	-	-	Chest	41.2	4
8	M	10y6m	-	-	Neck	31.44	3.2
9	M	5y9m	-	-	Right arm	46.88	2.6
10	F	10y9m	-	-	Left upper arm	103.47	4.4
11	M	3y11m	-	-	back	62.93	4.3
12	M	6y10m	-	-	Right upper arm	48.89	2.6
13	F	4y5m	-	-	Right upper arm	57.76	4.4
14	F	9y5m	-	-	Right shoulder	50.70	2.6
15	F	12y8m	-	-	Right forearm	50.6	3.4

TABLE S3. A list of antibodies used for the flow cytometry analysis of skin immune cells

Antigen	Clone	Fluorophore	Company	Product#	Host
**CD45**	30-F11	BUV563	BD	565710	Rat IgG
**CD4**	GK1.5	BUV496	BD	564667	Rat IgG
**cKit (CD117)**	2B8	BUV395	BD	564011	Rat IgG
**CD11b**	M1/70	BV786	BD	740861	Rat IgG
**TNFa**	MP6-XT22	BV711	Biolegend	506349	Rat IgG
**F4/80**	T45-2342	BV650	BD	743282	Rat IgG
**gdTCR**	GL3	BV605	BD	744116	Hamster IgG
**Siglec-F**	E50-2440	BV421	BD	562681	Rat IgG2a
**IL-4**	11B11	FITC/AF488	Biolegend	504111	Rat IgG
**IL-17A**	TC11-18H10.1	PE-Cy7	Biolegend	506921	Rat IgG
**CD3**	145-2C11	PE-CF594	BD	562332	Hamster igG
**CD123**	5B11	PE	Biolegend	106005	Rat IgG2a
**IFNg**	XMG1.2	APC-Cy7	BD	561479	Rat IgG
**CD8**	53-69.7	AF700	BD	557959	Rat IgG
**FceR1**	MAR-1	AF647	BioLegend	134309	Hamster IgG
**CD127**	A7R34	PE-Cy7	Biolegend	135013	Rat IgG
**Linage marker**	multi	AF700	Biolegend	133313	Multi
**CD25**	PC61	APC	Biolegend	102011	Rat IgG

Table S4. Primer sequences used for quantitative RT-PCR

Target	Primer sequence
**β-actin**	F: 5’-GCG CAA GTA CTC TGT GTG GA-3’
	R: 5’-GAA AGG GTG TAA AAC GCA GC-3’
**Dcir**	F: 5’-GAC TCG TCT TCA TGT ACC GTC T-3’
	R: 5’-AGC AAC AGA GAA TAA GAT TGC CA-3’
**IL-4**	F: 5’-GGT CTC AAC CCC CAG CTA GT-3’
	R: 5’-GCC GAT GAT CTC TCT CAA GTG AT-3’
**IL-13**	F: 5’-AAC GGC AGC ATG GTA TGG AGT G-3’
	R: 5’-TGG GTC CTG TAG ATG GCA TTG C-3’
**TNF-α**	F: 5’-GGT GCC TAT GTC TCA GCC TCT T-3’
	R: 5’-GCC ATA GAA CTG ATG AGA GGG AG-3’
**IL-33**	F: 5’-CTA CTG CAT GAG ACT CCG TTC TG-3’
	R: 5’-AGA ATC CCG TGG ATA GGC AGA G-3’

Table S5. Information about antibodies used for western blot (WB), flow cytometry (FC), and immunofluorescence staining (IF)

Target	Clone	Source	Use
6xHis-HRP	MAB050H	R&D Systems	ELISA (1:250)
β-actin	2F1-1	BioLegend	WB (1:1000)
CD107a	1D4B	BioLegend	FC (1:50)
c-Kit	2B8	BioLegend	FC (1:100)
DCIR	MAB2617 (ms)	Novus Biologicals	IF (1:200)
	9E8 (ms)	Invitrogen	FC (1:100)
	MAB1748 (hm)	Novus Biologicals	WB (1:500)
FcεRI	MAR-1	BioLegend	FC (1:100)
Ox-CaMKII	07-1387	Millipore Sigma	IF (1:200)
Tryptase	AF1937 (ms)	R&D Systems	IF (1:200)
	725445 (hm)	R&D Systems	IF (1:200)

REFERENCES

1. Alphonse MP, Rubens JH, Ortines RV, Orlando NA, Patel AM, Dikeman D, et al. Pan-caspase inhibition as a potential host-directed immunotherapy against MRSA and other bacterial skin infections. *Science translational medicine.* 2021;13(601).
2. Hu X, Shen Y, Zhao Y, Wang J, Zhang X, Tu W, et al. Epithelial Aryl Hydrocarbon Receptor Protects From Mucus Production by Inhibiting ROS-Triggered NLRP3 Inflammasome in Asthma. 2021;12(4810).
3. Zhang Y, Do DC, Hu X, Wang J, Zhao Y, Mishra S, et al. CaMKII oxidation regulates cockroach allergen-induced mitophagy in asthma. *The Journal of allergy and clinical immunology.* 2021;147(4):1464-77 e11.

Suppl figure 1

A

Th1/Th2/Th17 and mast cells

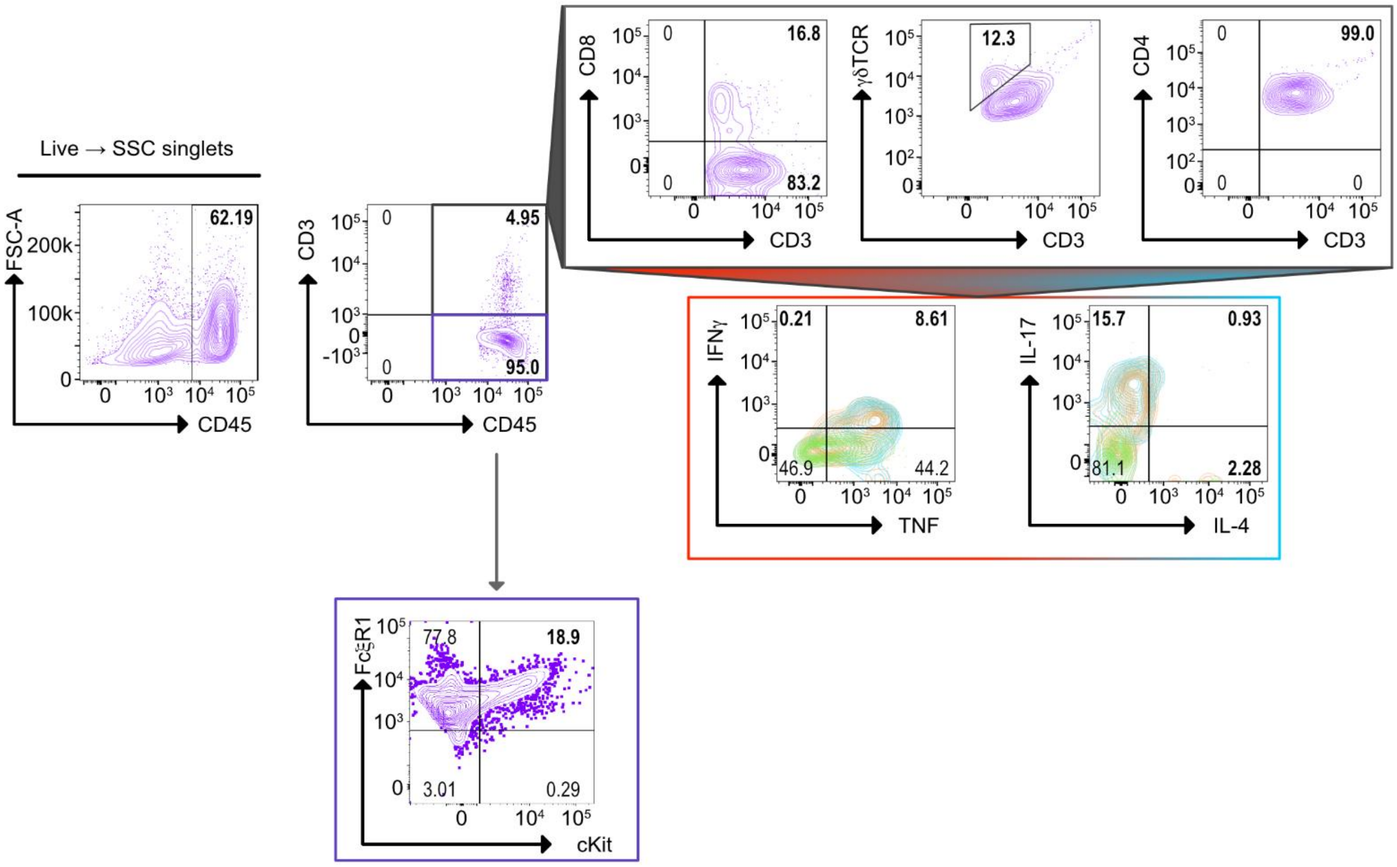

B

ILC2

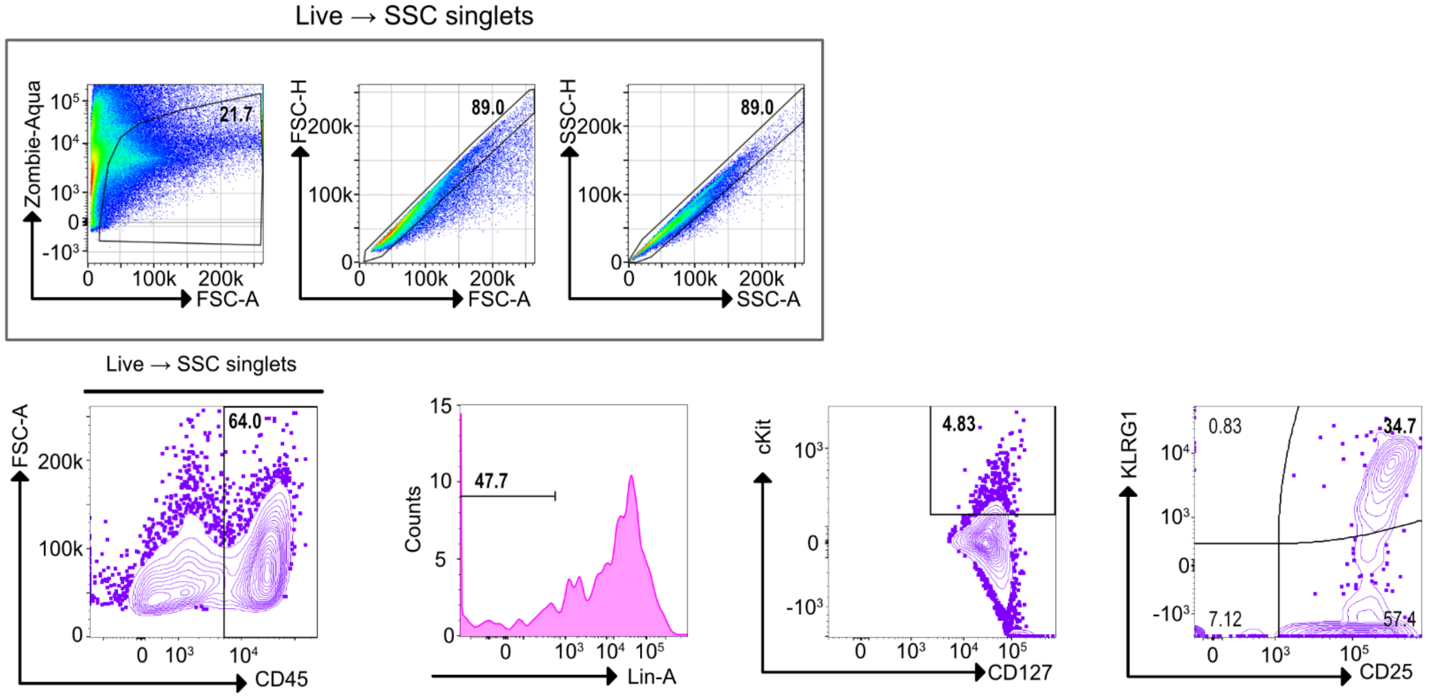

Suppl figure 2

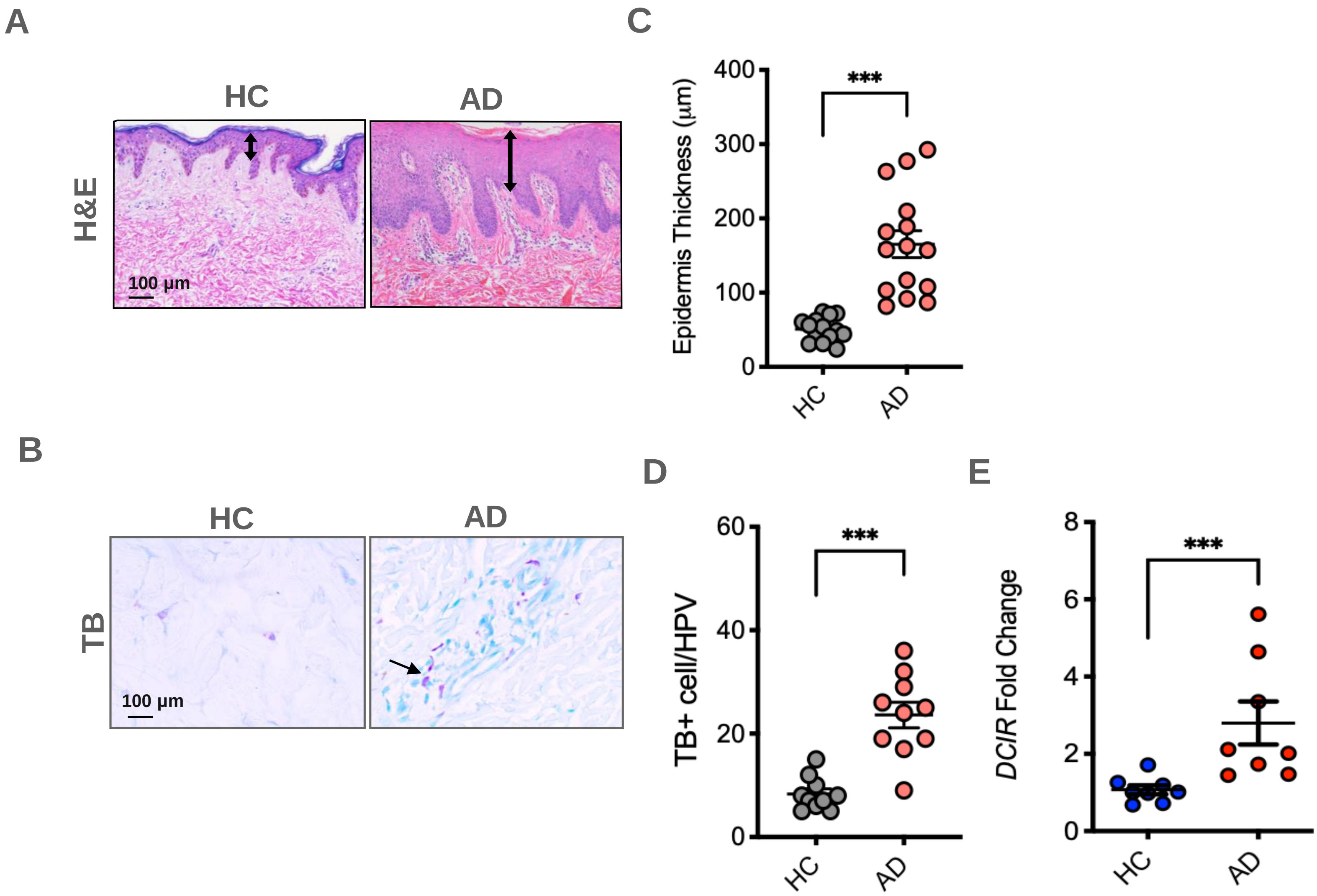

Suppl figure 3

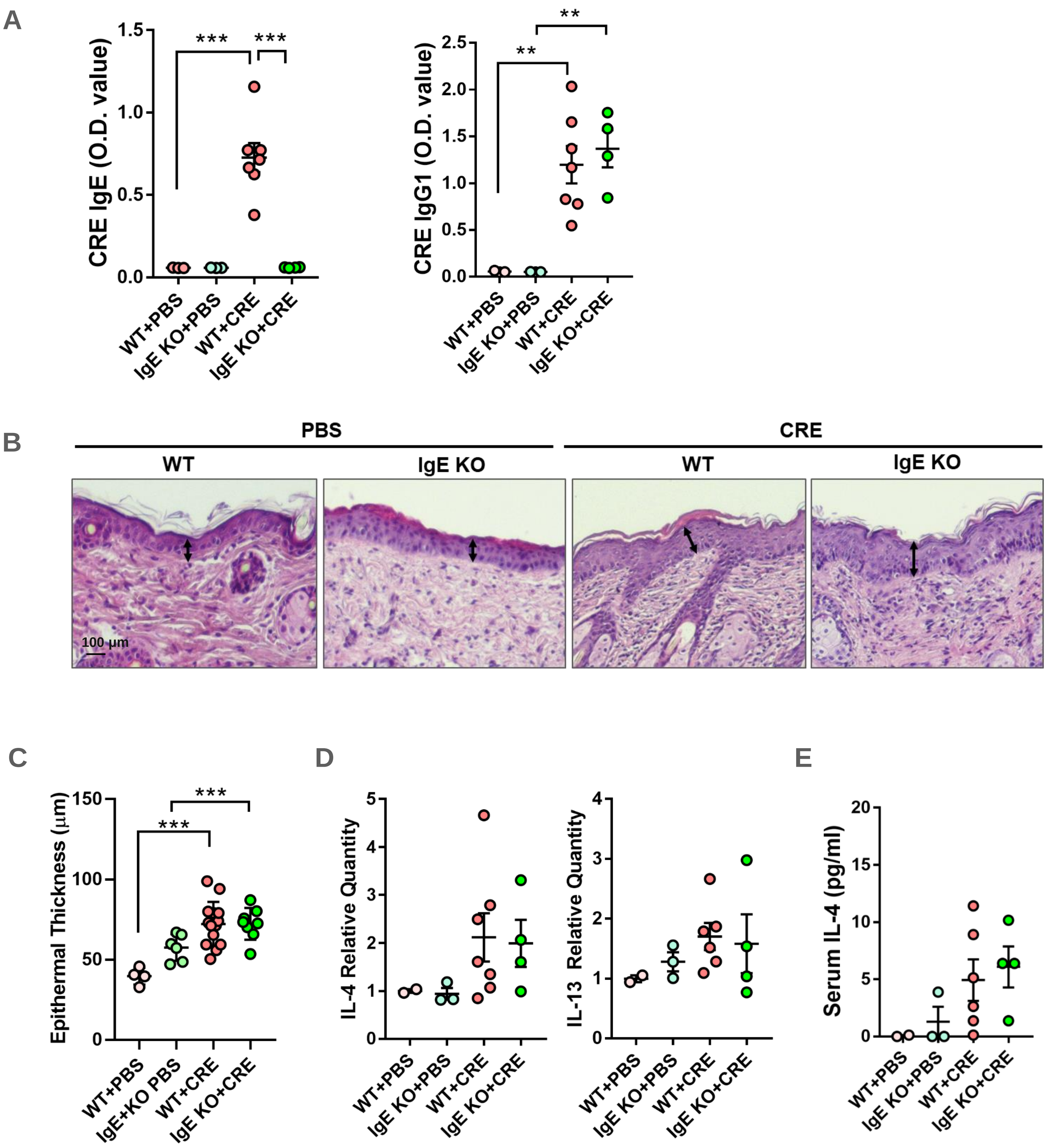

Suppl figure 4

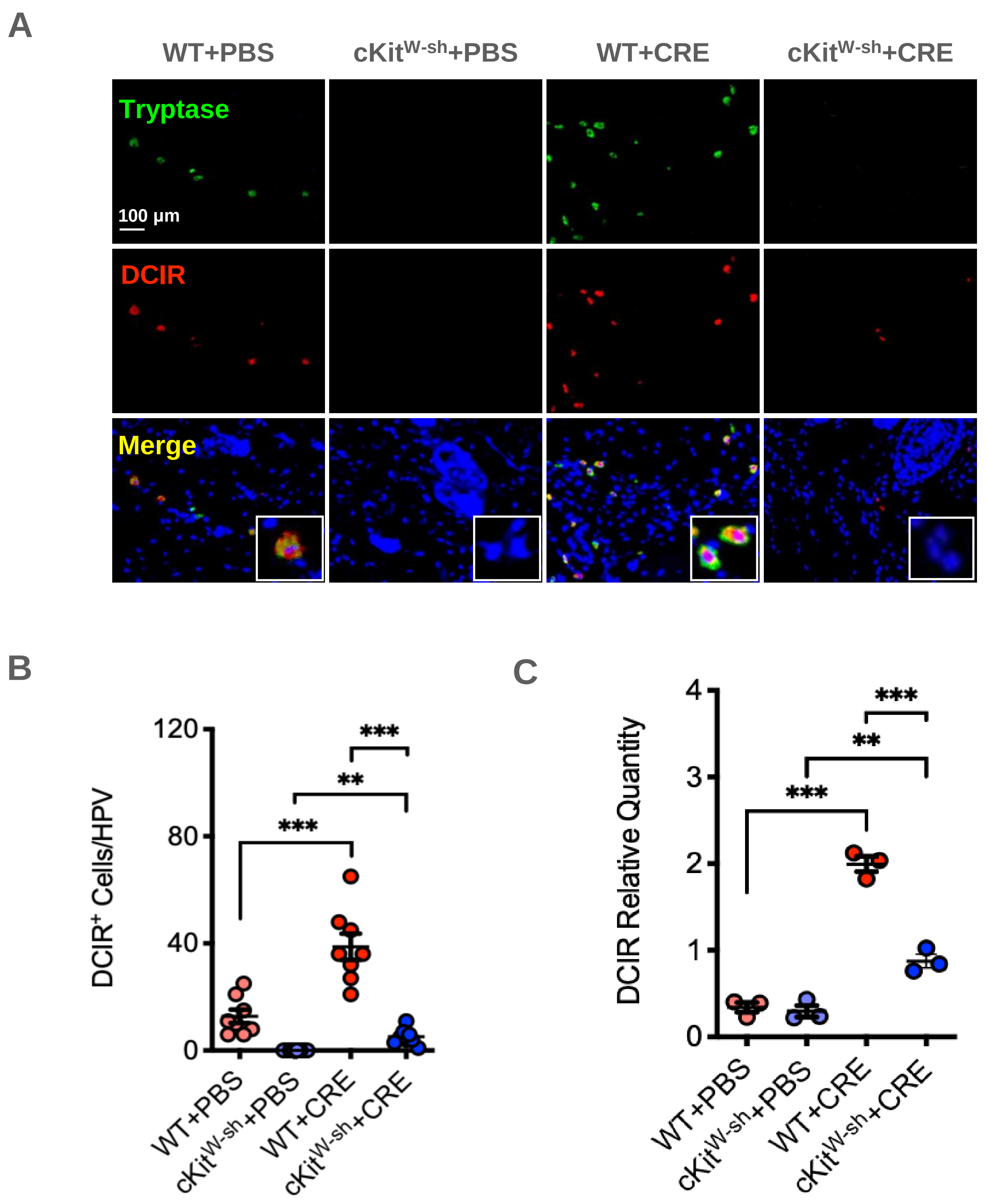

Suppl figure 5

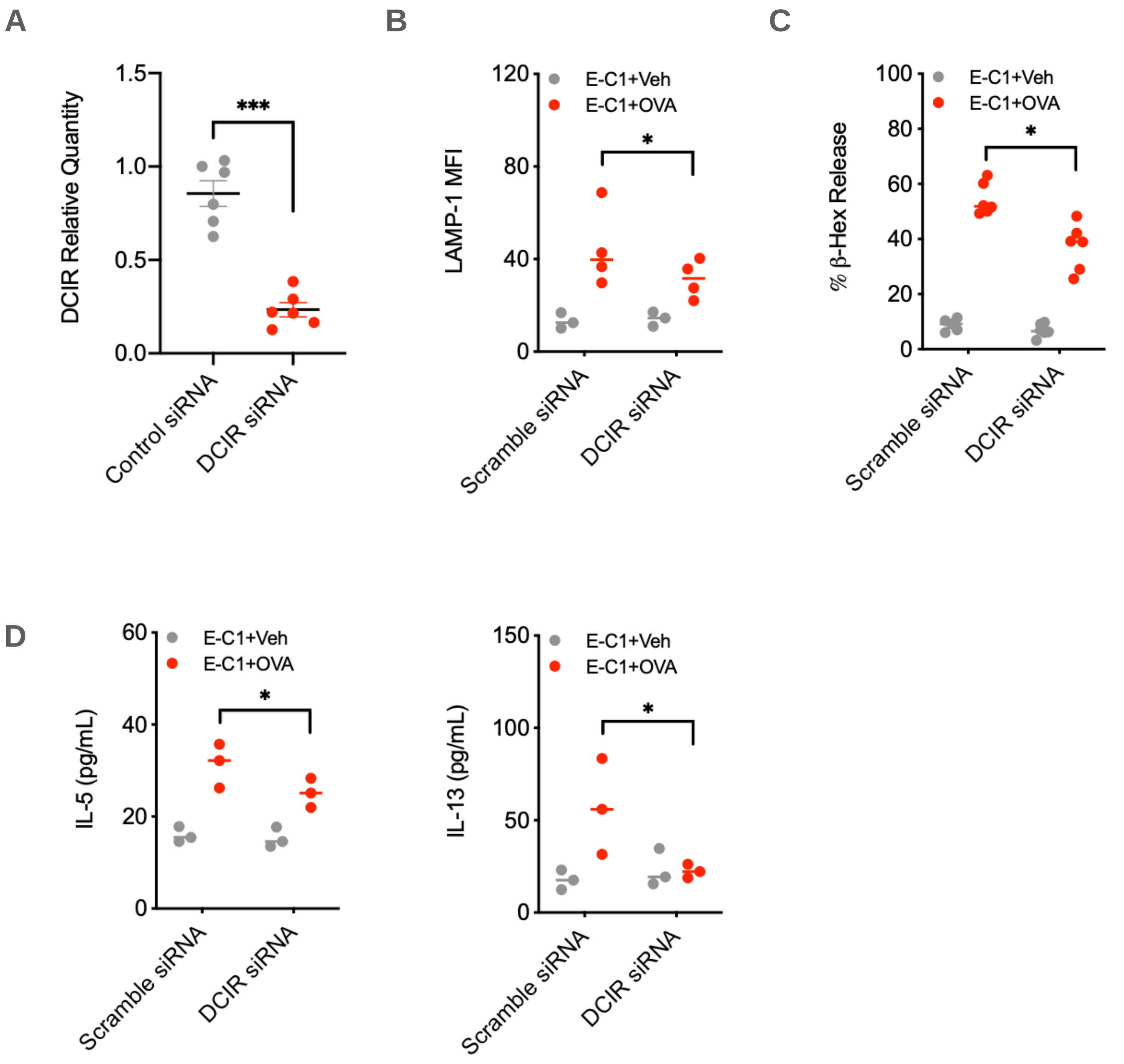

Suppl figure 6

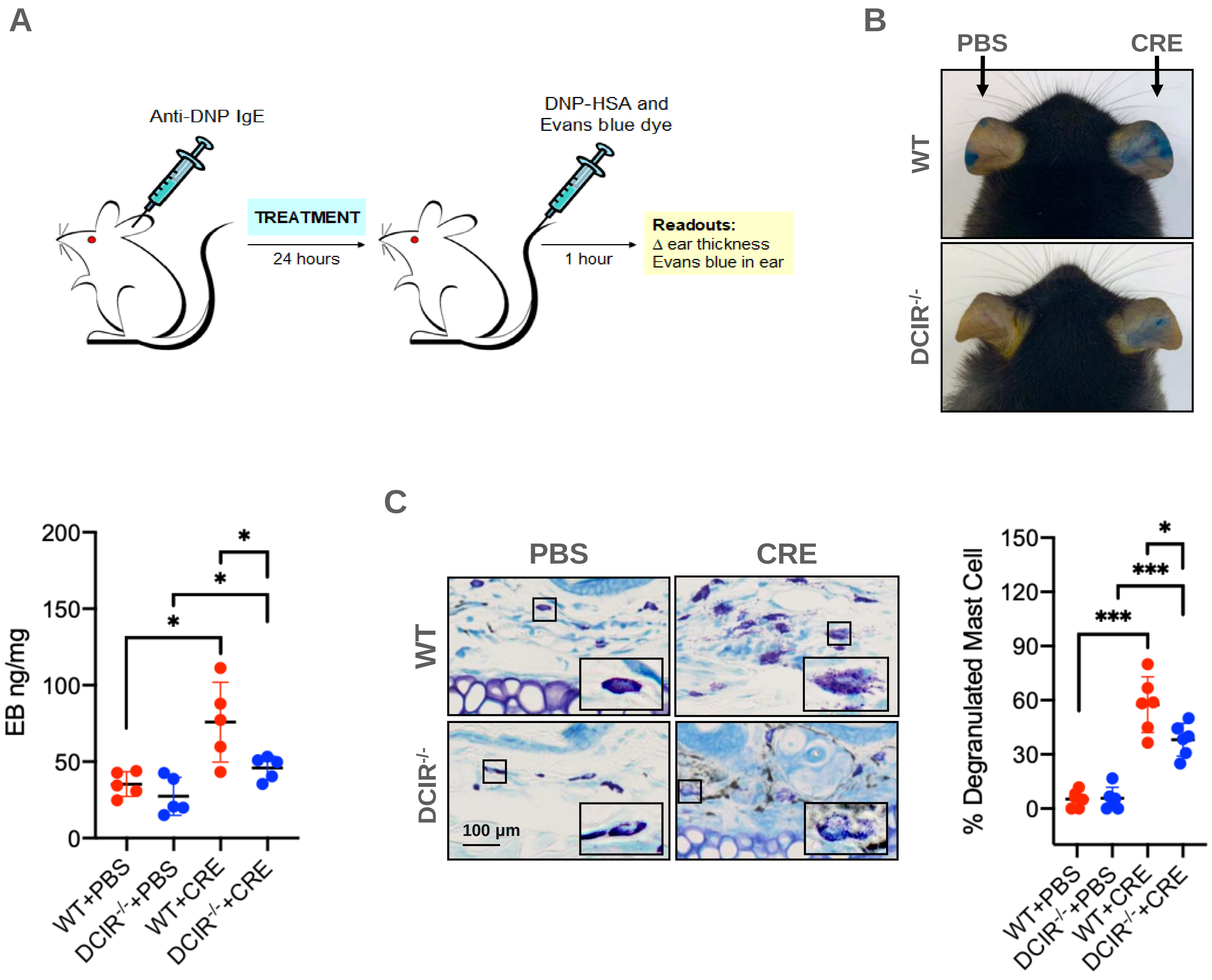

Suppl figure 7

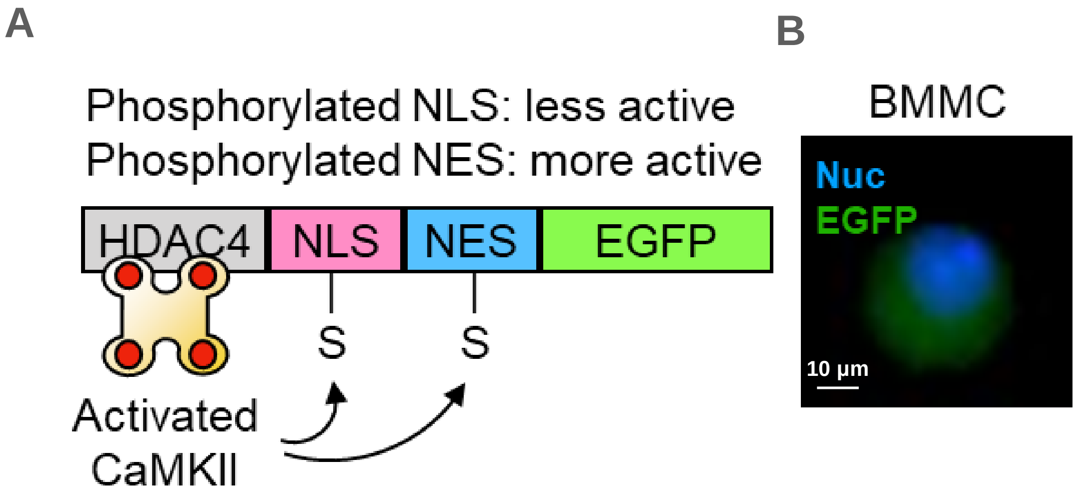

Supplement: Supplemental data [file jciinsight-7-152559-s170.pdf]
